# A Role for Macro-ER-Phagy in ER Quality Control

**DOI:** 10.1371/journal.pgen.1005390

**Published:** 2015-07-16

**Authors:** Zhanna Lipatova, Nava Segev

**Affiliations:** Department of Biochemistry and Molecular Genetics, University of Illinois at Chicago, Chicago, Illinois, United States of America; Yale University, UNITED STATES

## Abstract

The endoplasmic-reticulum quality-control (ERQC) system shuttles misfolded proteins for degradation by the proteasome through the well-defined ER-associated degradation (ERAD) pathway. In contrast, very little is known about the role of autophagy in ERQC. Macro-autophagy, a collection of pathways that deliver proteins through autophagosomes (APs) for degradation in the lysosome (vacuole in yeast), is mediated by autophagy-specific proteins, Atgs, and regulated by Ypt/Rab GTPases. Until recently, the term ER-phagy was used to describe degradation of ER membrane and proteins in the lysosome under stress: either ER stress induced by drugs or whole-cell stress induced by starvation. These two types of stresses induce micro-ER-phagy, which does not use autophagic organelles and machinery, and non-selective autophagy. Here, we characterize the macro-ER-phagy pathway and uncover its role in ERQC. This pathway delivers 20–50% of certain ER-resident membrane proteins to the vacuole and is further induced to >90% by overexpression of a single integral-membrane protein. Even though such overexpression in cells defective in macro-ER-phagy induces the unfolded-protein response (UPR), UPR is not needed for macro-ER-phagy. We show that macro-ER-phagy is dependent on Atgs and Ypt GTPases and its cargo passes through APs. Moreover, for the first time the role of Atg9, the only integral-membrane core Atg, is uncoupled from that of other core Atgs. Finally, three sequential steps of this pathway are delineated: Atg9-dependent exit from the ER en route to autophagy, Ypt1- and core Atgs-mediated pre-autophagsomal-structure organization, and Ypt51-mediated delivery of APs to the vacuole.

## Author Summary

ER-quality control (ERQC) ensures delivery of “native” proteins through the secretory pathway. Currently, ER-associated degradation (ERAD), which delivers misfolded proteins for degradation by the proteasome, is considered a major ERQC pathway, with autophagy as its backup. Until now, the role of autophagy, which shuttles cellular components for degradation in the lysosome through autophagosomes (APs), in ERQC was ill defined. Recently, the process of ER degradation induced by ER stress was defined as micro-ER-phagy, which does not require autophagic machinery and does not pass through APs. Here, we characterize the macro-ER-phagy pathway, which delivers excess membrane proteins for degradation in the lysosome, as a novel ERQC pathway. This pathway functions in the absence of cellular or ER stress and can be further induced by overexpression of a single integral-membrane protein. Unlike the micro-ER-phagy pathway, the marco-ER-phagy pathway requires core autophagy-specific proteins, Atgs, and Ypt/Rab GTPases. In addition, for the first time, the function of the only membrane core Atg, Atg9, was uncoupled from that of the other core Atgs. Whereas Atg9 plays a role in the assembly of ER-to-autophagy membranes (ERAM), other core Atgs and Ypt1 assemble the Atg-protein complex on ERAM to form the pre-autophagosomal structure.

## Introduction

One third of all newly synthesized proteins enter the endoplasmic reticulum (ER). However, only a small fraction is transported to their final destination. A large fraction (30–75%) fails to fold and mature properly, does not pass the ER quality control (ERQC) and gets degraded [1]. Two different cellular pathways shuttle proteins from the ER for degradation: ER associated degradation (ERAD) and autophagy. Whereas the importance of ERAD in ERQC has been studied extensively and is well established, not much is known about the role of autophagy in ERQC [2].

ERAD delivers proteins from the ER for degradation by the cytoplasmic proteasome. ERAD substrates include soluble and integral-membrane proteins that fail to fold properly or assemble into complexes. Substrate recognition happens in the lumen or the membrane of the ER by chaperones (e.g., BiP). These substrates are translocated back to the cytoplasm where they are ubiquitinated and degraded by the proteasome [3,4]. Under conditions that stimulate accumulation of misfolded proteins (e.g., DTT and tunicamycin, inhibitors of disulfide-bond formation and glycosylation, respectively), ER stress and the conserved unfolded-protein response (UPR) are induced. In yeast, UPR induction requires two proteins, the endonuclease Ire1 and the transcription factor Hac1, which binds to UPR elements and stimulates the transcription of ERAD machinery components [5]. Multiple human disorders have been associated with ERAD [2].

In autophagy, cargo is delivered for degradation in the lysosome (vacuole in yeast), a major recycling cellular compartment. There are three major types of autophagy: macro, micro and chaperone mediated (CMA) [6]. Macro-autophagy, the best studied type, is a collection of cellular degradation pathways in which cargo is engulfed by a double-membrane organelle termed the autophagosome (AP) that fuses with the lysosome. All macro-autophagy pathways start with the formation of the pre-autophagosomal structure (PAS), which is mediated by the core autophagy-related proteins (Atgs). PAS includes subunits of the Atg protein complex and membranes; the latter are thought to be supplied by the only integral-membrane core Atg, Atg9 [7,8]. Macro-autophagy can be nonselective, when induced by stress, or selective, e.g., cytoplasm-to-vacuole transport (CVT), mitophagy (autophagy of mitochondria), pexophagy (autophagy of peroxisomes) [8], and ER-phagy (autophagy of the ER), which is discussed here.

Micro-autophagy and CMA, about which less is known, do not require Atgs and their cargos are not delivered through APs [6]. In micro-autophagy, cargo enters the lysosome through invagination of its membrane. For example, under certain growth conditions, peroxisome clusters can enter the lysosome via micro-pexophagy [9]. Likewise, dispensable portions of the nucleus can be delivered into the lysosome via the piecemeal micro-autophagy of the nucleus [10]. CMA, which has been described so far only in mammalian cells, is highly specific and involves translocation of unfolded proteins through the lysosomal membrane [6].

Until recently, the terms ER-phagy and reticulophagy, autophagy of the ER, have been loosely used to describe any process that delivers ER for degradation in the lysosome. For example, ER was identified as one of multiple membrane sources for AP biogenesis [11]. In addition, during prolonged ER stress, nonselective macro-autophagy can be induced [12], and was suggested to serve as a backup for ERAD [4,13]. Most notably, ER-phagy induced by ER stress [14], was recently characterized as micro-ER-phagy, which does not require the macro-autophagy machinery components [15]. Thus, almost nothing is currently known about selective macro-autophagy of the ER [16]. We have recently used the term ER-phagy to describe a Ypt1 GTPase-dependent pathway through which an overexpressed membrane protein is delivered to the vacuole for degradation [17]. The major point of that paper was to clarify that accumulation of GFP-Snc1, a marker traditionally used for endosome-to-Golgi transport, in mutant cells defective in Ypt1 function is not due to a defect in endosome-to-Golgi transport, but, rather to its role in autophagy emanating from the ER. However, under what conditions this pathway is induced, the nature of its cargos and the role of core Atgs in it, are not known. This pathway is defined here as macro-ER-phagy.

The eleven yeast Ypt GTPases and their seventy Rab homologues regulate and coordinate the multiple intra-cellular trafficking pathways [18]. These GTPases are activated by their guanine-nucleotide exchange factors (GEFs) to recruit their various effectors, which in turn mediate vesicular transport steps [19,20]. At least three Ypt GTPases were implicated in regulation of autophagy. We have shown that Ypt1, a Rab1 homolog, coordinates the first steps of two different pathways that emanate from the ER: secretion and autophagy [21,22]. Ypt1 does this in the context of two distinct GTPase modules that contain different GEFs and effectors [23]. Vps21, a Rab5 homolog, regulates two different pathways, endocytosis and autophagy, in the context of the same module [24,25]. Finally, Ypt7, a Rab7 homolog, regulates fusion of APs with the vacuole [26].

We have previously shown that components of the autophagy-specific Ypt1 module, including the Trs85-containing TRAPP III GEF, Ypt1-GTPase, and the Atg11 effector, regulate delivery of an overexpressed integral plasma membrane (PM) protein from the ER to the vacuole for degradation. The membrane protein we used was GFP-tagged Snc1-PEM; a variant of the vSNARE Snc1 that cannot be internalized by endocytosis [27]. We used the term ER-phagy for this pathway [22]. However, especially in light of recent characterization of drug-induced ER-phagy as micro-ER-phagy, it is crucial to determine if and how ER-phagy induced by overexpression of an integral-membrane protein is different from other ER-phagy processes described recently, e.g., micro-ER-phagy and backup for ERAD. Here, we characterize this pathway as macro-ER-phagy, and determine that it plays a role in vacuolar recycling of some ER-resident proteins even under normal growth conditions and can be further induced by overexpression of a single integral-membrane protein. We also show that this pathway requires core Atgs, and identify its cargos. Moreover, we define three sequential steps in this pathway, which are dependent on Atg9, Ypt1 and core Atgs, and Vps21.

Budding yeast was instrumental for current conceptual understanding of intracellular trafficking [28], autophagy [8,29], and the role of Ypt/Rab GTPases in these processes [30] in human cells. Moreover, machinery components that mediate these processes are conserved from yeast to humans and are relevant to health and disease. For example, the Ypt1 human homolog hRab1A was defined recently as an oncogene [31]. Therefore, there is no doubt that the principles of macro-ER-phagy characterized here would pertain to human cells. The potential relevance of clearance of excess membrane proteins by macro-ER-phagy to human disease is discussed.

## Results

### Macro-ER-phagy requires core autophagy machinery

We have previously shown that mutations in components of the autophagy-specific Ypt1 GTPase module, *ypt1-1*, *trs85∆*, and, *atg11∆*, are defective in delivery of overexpressed GFP-Snc1-PEM, a PM integral membrane protein, from the ER to the vacuole for degradation. We termed this pathway ER-phagy [17]. Because here we identify this pathway as macro-ER-phagy, and to distinguish it from the recently characterized micro-ER-phagy [15], we will hereinafter use the term macro-ER-phagy.

To identify other autophagy machinery components involved in macro-ER-phagy, we determined whether deletions of several Atgs result in a defect in delivering overexpressed GFP-Snc1-PEM to the vacuole through this pathway, using the following three criteria: increase in the protein level of GFP-Snc1-PEM, its accumulation in aberrant ER structures, and induction of UPR. These aberrant structures were previously identified as a cluster of ER-derived membrane-bound vesicles using immune-electron microscopy and anti-Hmg1 antibodies [17]. Their molecular composition is further characterized below.

**Fig 1:**
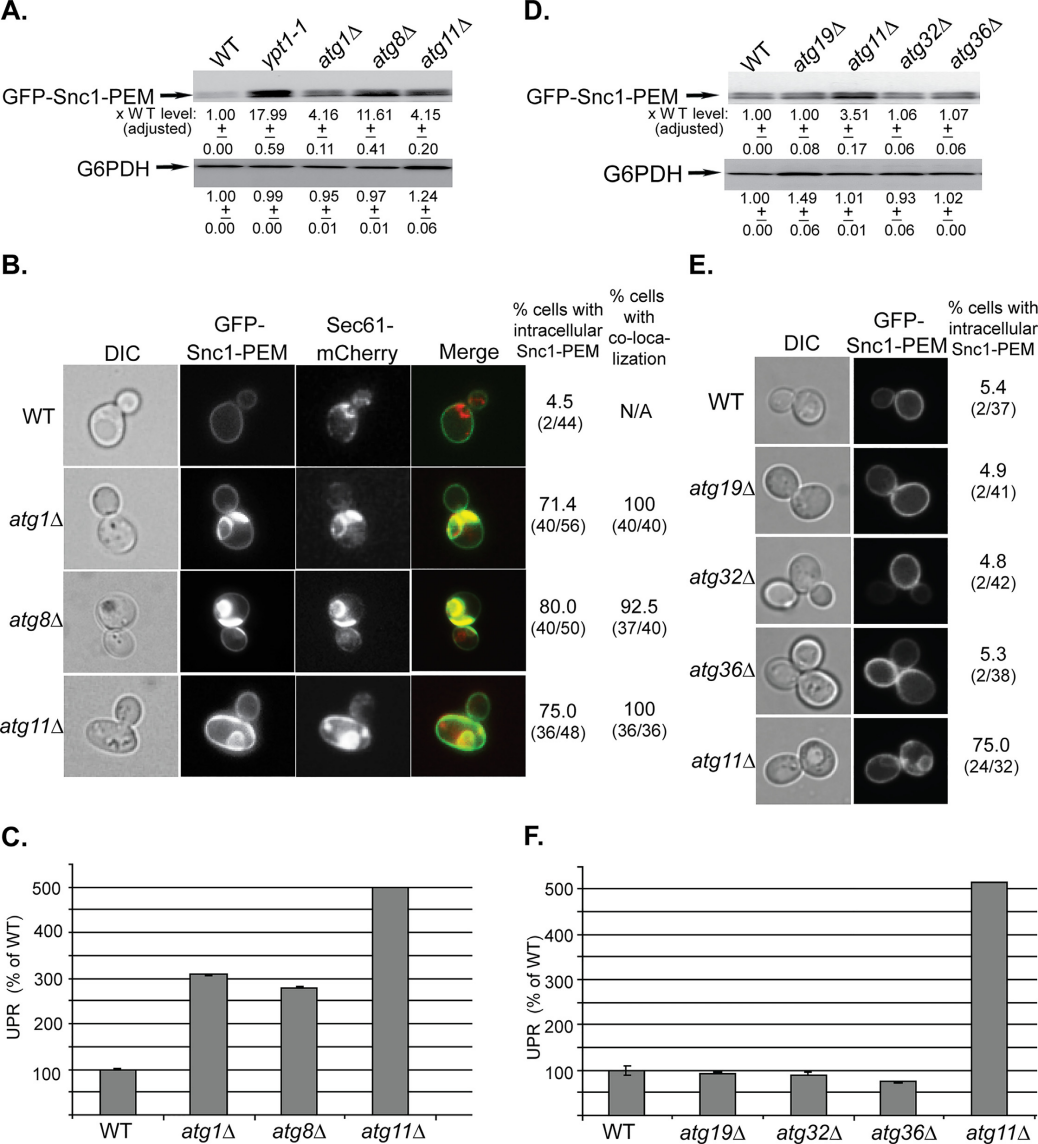
The general Atgs Atg1 and Atg8 and the selective Atg Atg11, but not other selective Atgs, are required for macro-ER-phagy. **A.** GFP-Snc1-PEM protein accumulates in *atg1∆*, *atg8∆*, and *atg11∆* mutant cells. Lysates were prepared from wild type (WT), *ypt1-1* (for comparison), *atg1∆*, *atg8∆*, and *atg11∆* mutant cells transformed with a 2μ plasmid expressing GFP-Snc1-PEM from the TPI promoter. The level of GFP-Snc1-PEM was determined using immunoblot analysis with anti-GFP antibodies. The bands were quantified and increase in the protein level in mutant versus the WT cells is shown under the blot and adjusted to the G6PDH loading control. **B.** GFP-Snc1-PEM protein accumulates in aberrant ER structures in *atg1∆*, *atg8∆*, and *atg11∆* mutant cells. The ER-marker Sec61 was tagged with mCherry in strains from panel A, and the cells were examined by live-cell microscopy. Shown from left to right: DIC, GFP, mCherry, merge, % cells with intracellular GFP-Snc1-PEM (number of cells with internal GFP-Snc1-PEM / number of cells visualized), and % cells in which intra-cellular Snc1-PEM co-localizes with Sec61. **C.** UPR is induced in *atg1∆*, *atg8∆*, and *atg11∆* mutant cells. Cells from panel A were transformed with a second plasmid that expresses β-gal from a UPR promoter. UPR was determined and expressed as % of the WT response. **D-E.** Unlike deletion of Atg11, deletion of other known selective Atgs required for the CVT pathway (Atg19), mitophagy (Atg32) and pexophagy (Atg36), does not result in increase of GFP-Snc1-PEM protein level (**D**), intra-cellular accumulation of GFP-Snc1-PEM, (**E**), and induction of the UPR response (**F**). Wild type (WT), *atg19∆*, *atg11∆*, *atg32∆*, and *atg36∆* mutant cells overexpressing GFP-Snc1-PEM were analyzed as described for panels A-C, respectively. **E.** Shown from left to right: DIC, GFP, and % cells with intracellular Snc1-PEM structures. +/- and error bars represent STDEV. Results in this figure represent at least two independent experiments.

Like Atg11, deletion of the two core autophagy components, Atg1 and Atg8, result in an increase in the GFP-Snc1-PEM protein level (Fig 1A), accumulation of aberrant Snc1-PEM intracellular structures that co-localize with the ER marker Sec61 (Fig 1B), and induction of UPR (Fig 1C). While >70% of all three mutant cells accumulate aberrant GFP-Snc1-PEM intracellular structures, the levels of protein accumulation and UPR induction vary, and are lower than those observed in *ypt1-1* mutant cells (see S1A Fig). Deletion of another core autophagy component, Atg17, by itself had no effect on macro-ER-phagy. However, the double mutant *atg11∆ atg17∆* displayed more severe defects in all three assays than those of the *atg11∆* alone, similar to that of the *ypt1-1* mutation (S1A–S1C Fig). More severe defects in other autophagy types were previously observed for the double *atg11∆ atg17∆* mutant than for the single deletions [32]. These results indicate that the role of Ypt1 in macro-ER-phagy is mediated by both Atg11-dependent and-independent GTPase modules.

Atg11 plays a role in all selective autophagy processes [33]. To determine whether other selective-autophagy Atgs play a role in macro-ER-phagy, we analyzed the effect of deletions of Atg19, Atg32, and Atg36, which are required for CVT, mitophagy and pexophagy, respectively, on delivery of overexpressed GFP-Snc1-PEM to the vacuole [33]. In all three assays, *atg19∆*, *atg32∆* and *atg36∆* mutant cells behaved like wild-type cells (Fig 1D–1F). However, when combined with *atg11∆*, all three double mutant cells behaved like *atg11∆* single mutant cells (S1D–S1F Fig).

Together, these data indicate that macro-ER-phagy requires the core autophagy machinery, but not any of the known selective autophagy components other than Atg11.

### Atg9 is required for macro-ER-phagy in a step before other PAS organizers

**Fig 2:**
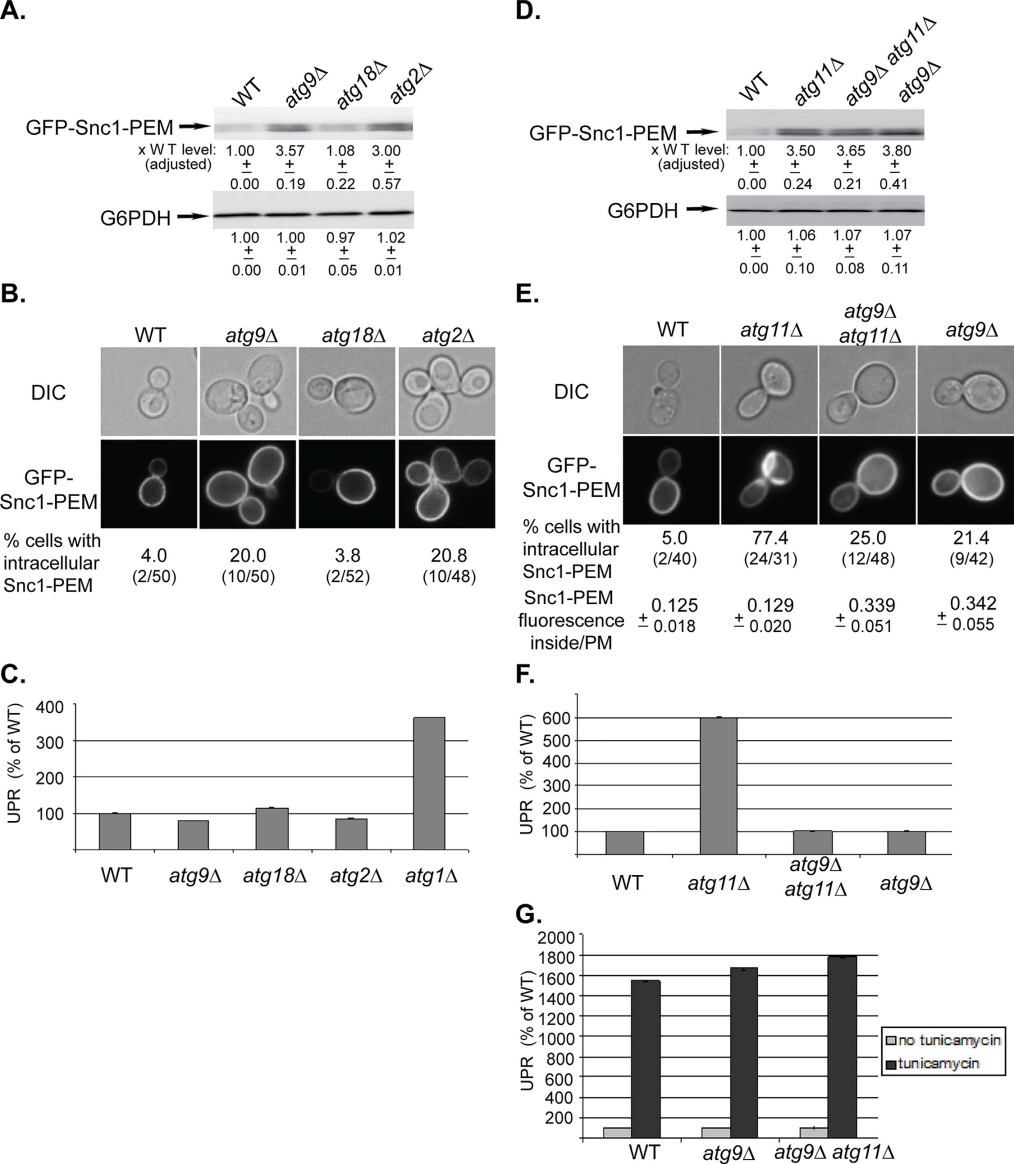
Depletion of Atg9 and Atg2, but not Atg18, result in a macro-ER-phagy phenotype different from that of other core Atgs, and *atg9∆* is epistatic to *atg11∆*. **A-C**. While the level of overexpressed GFP-Snc1-PEM is increased in atg9∆ and atg2∆, but not atg18∆, mutant cells, it does not accumulate in aberrant structures and does not induce UPR. Wild type (WT), *atg9∆*, *atg18∆*, and *atg2∆* mutant cells overexpressing GFP-Snc1-PEM were analyzed as described for Fig 1A–1C, respectively. The tested phenotypes: the level of GFP-Snc1-PEM protein (**A**), accumulation of GFP-Snc1-PEM in aberrant structures (**B**), and induction of the UPR response (**C,** *atg1∆* is shown as a positive control). **B.** Shown from top to bottom: DIC, GFP, and % cells with intracellular Snc1-PEM structures. **D-G.** Atg9 is epistatic to Atg11 in macro-ER-phagy. *ATG9* was deleted in wild type and *atg11∆* mutant cells and the effects of overexpression of GFP-Snc1-PEM were determined in the single and double mutants as described in Fig 1 legend. **D.** Deletion of *ATG9* in wild type (WT) or *atg11∆* mutant cells results in an increase of GFP-Snc1-PEM protein level similar to the increase in *atg11∆* mutant cells. **E.** Deletion of *ATG9* in wild type or *atg11∆* mutant cells results in an increase of intracellular GFP-Snc1-PEM fluorescence. However, only ~20% of the *atg9∆* single-, and *atg9∆ atg11∆* double-mutant cells accumulate GFP-Snc1-PEM in aberrant structures, as compared with ~75% of *atg11∆* mutant cells. Shown from top to bottom: DIC, GFP, % cells with aberrant intracellular GFP-Snc1-PEM structures, ratio of GFP-Snc1-PEM fluorescence inside/PM (30 cells were analyzed for each strain). **F.** UPR is induced in *atg11∆*, but not in *atg9∆* single- and *atg9∆ atg11∆* double-mutant cells overexpressing GFP-Snc1-PEM. **G.** UPR can be induced in *atg9∆* single-, and *atg9 atg11∆* double-mutant cells overexpressing GFP-Snc1-PEM by tunicamycin. +/- and error bars represent STDEV. Results in this figure represent at least two independent experiments.

Atg9, the only integral-membrane core Atg protein, is required for PAS formation in all autophagy processes [34]. Analysis of the *atg9∆* effect on delivery of overexpressed GFP-Snc1-PEM to the vacuole revealed a phenotype different from that of mutations in other core Atgs and Ypt1 (shown in Fig 1). The level of GFP-Snc1-PEM protein was >3-fold higher than its level in WT cells, comparable to the increase in *atg11∆* mutant cells (Fig 2A and 2D). A 2.5-fold increase was also observed in the cytoplasmic fluorescence of GFP-Snc1-PEM (S2A Fig). However, whereas ~75% of *atg11∆* mutant cells accumulate GFP-Snc1-PEM in large intracellular structures, only 20% of the *atg9∆* mutant cells contain small intracellular GFP-Snc1-PEM structures (Fig 2B and 2E), which co-localize with the ER marker Sec61-mCherry (S2A Fig). Moreover, UPR was not induced in *atg9∆* mutant cells (Fig 2C and 2F). In addition, while overexpression of GFP-Snc1-PEM had no effect on the growth WT or *atg11∆* mutant cells, it caused a growth defect in *atg9∆* mutant cells (S2B Fig).

Two other Atgs, Atg2 and Atg18, affect the function of Atg9 by mediating its recycling from APs to peripheral sites [35]. The effects of the *atg2∆* and *atg18∆* mutations on macro-ER-phagy were determined. Whereas *atg2∆* showed phenotypes similar to those of *atg9∆*, *atg18∆* showed no effect (Fig 2A–2C). Different effects of Atg2 and Atg18 on autophagy were previously observed in Drosophila [36].

The different macro-ER-phagy phenotype of *atg9∆* mutant cells allowed us to perform epistasis analyses with *atg11∆*, *atg1∆*, and *ypt1-1*. The level of GFP-Snc1-PEM in the double mutants *atg9∆ atg11∆* and *atg9∆ atg1∆* is similar to its level in the single mutants (Figs 2D and S2C, respectively). The fact that the level does not increase in the double mutant indicates that Atg9 functions in the same pathway as Atg11 and Atg1. Importantly, both the microscopy and the UPR assays show that the *atg9∆* mutation “masks” the phenotypes of *atg11∆* and *atg1∆*. Specifically, whereas >75% of *atg11∆* and *atg1∆* single-mutant cells accumulate large intracellular GFP-Snc1-PEM structures, small structures were observed only in <25% the double mutants, *atg9∆ atg11∆* and *atg9∆ atg1∆*, similar to the phenotype of the single *atg9∆* mutation (Figs 2E and S2D). Likewise, whereas UPR is induced in *atg11∆* and *atg1∆* single-mutant cells, it is not induced in *atg9∆ atg11∆* and *atg9∆ atg1∆* double mutants, similar to the phenotype of the single *atg9∆* mutation (Figs 2F and S2E). Both *atg9∆* and *atg9∆ atg11∆* mutant cells are not defective in UPR induction, as UPR can be induced in these cells by tunicamycin (Fig 2G).

**Fig 3:**
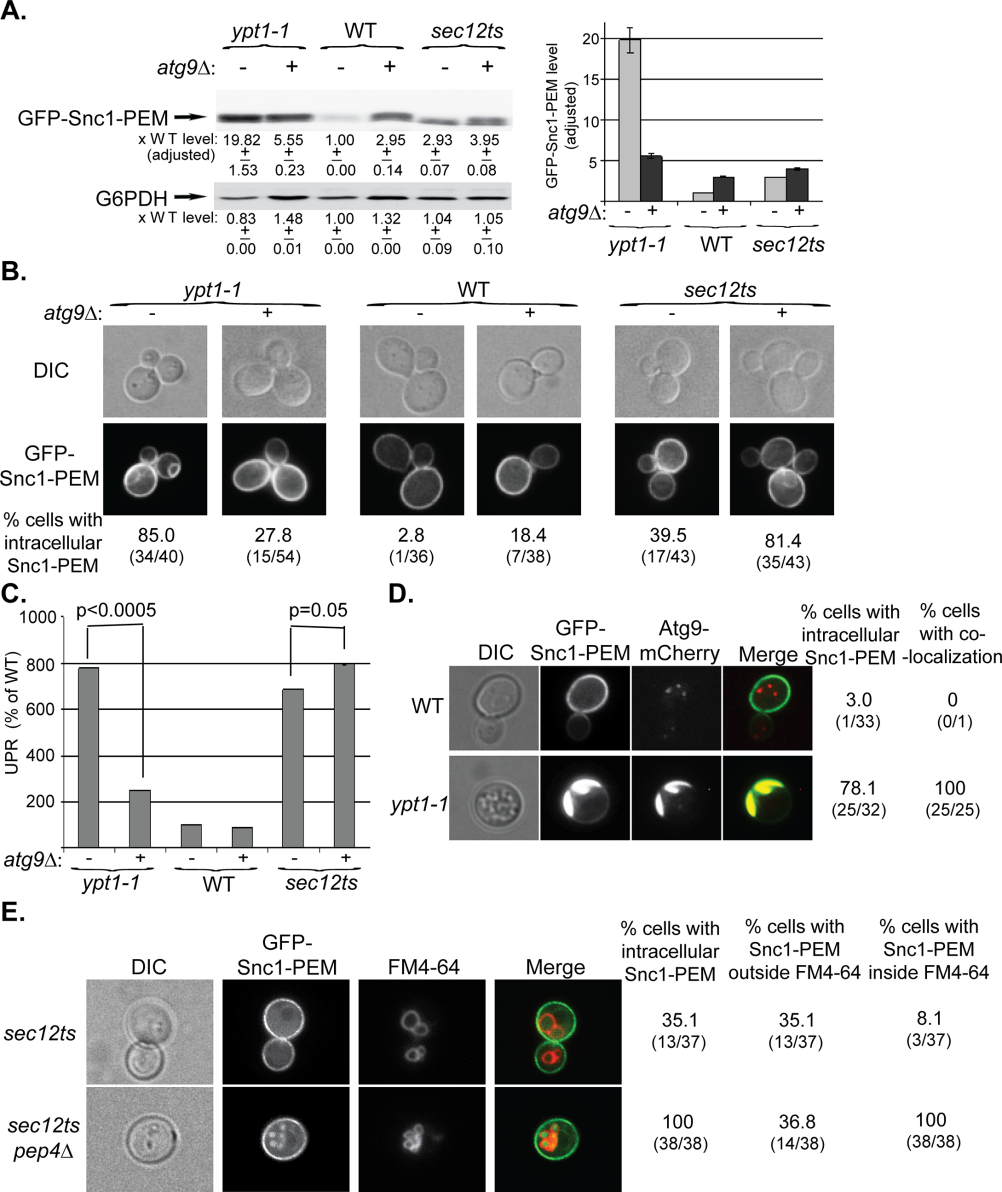
Atg9 is epistatic to Ypt1 in macro-ER-phagy and not to the ER-exit regulator Sec12, which is not defective in this process. **A-C.** *ATG9* was deleted in *ypt1-1*, WT and *sec12ts* mutant cells and the effect of overexpression of GFP-Snc1-PEM was determined in single and double mutant cells. Experiments were performed as described for Fig 1A–1C, respectively. **A.** Snc1-PEM is increased ~3 fold in *atg9∆* and *sec12ts* as compared to ~20 fold in *ypt1-1* mutant cells. Importantly, *atg9∆* is epstatic to the *ypt1-1*, but not to the *sec12ts*, mutation. Left, immuno-blot analysis, increase of the protein level in mutant versus the WT cells is shown under the blot; right, a bar graph summarizing the quantified data. **B.** Whereas deletion of *ATG9* in *ypt1-1* mutant cells results in three fold lower accumulation of GFP-Snc1-PEM in aberrant structures (85% to 27%), its deletion in *sec12ts* mutant cells results in two fold increased accumulation (~40% to ~80%). Shown from top to bottom: DIC, GFP, and % cells with intracellular Snc1-PEM structures. **C.** Deletion of *ATG9* in *ypt1-1* mutant cells overexpressing Snc1-PEM results in ~3.5 lower UPR (p-value<0.0005), but a slightly increased UPR in *sec12ts* mutant cells (p-value = 0.05). **D.** Atg9 is present on aberrant ER structures that accumulate in *ypt1-1* mutant cells. WT and *ypt1-1* mutant cells expressing Atg9-mCherry from the chromosome and GFP-Snc1-PEM from a 2μ plasmid were analyzed by live-cell microscopy. Whereas in WT cells (top), the GFP-Snc1-PEM localizes to the cell membrane and Atg9-mCherry to intracellular puncta, the two proteins co-localize in 100% of the *ypt1-1* mutant cells (bottom) that accumulate intracellular GFP-Snc1-PEM structures (~80%). Shown from left to right: DIC, GFP, mCherry, merge, % cells with intracellular Snc1-PEM, and % cells with co-localization (number of cells with observed phenotype / total number of cells analyzed). **E.** GFP-Snc1-PEM is delivered to the vacuole for degradation in *sec12ts* mutant cells. Accumulation of GFP-Snc1-PEM in vacuoles of *sec12ts* mutant cells, with and without deletion of the vacuolar protease Pep4, was determined using the FM4-64 dye, which labels the vacuolar membrane. Deletion of *PEP4* in *sec12ts* mutant cells results in an increase percent of cells in which GFP-Snc1-PEM accumulates inside the vacuole (from 8% to 100%). Shown from left to right: DIC, GFP, FM4-64 (vacuolar membrane), merge, % cells with aberrant GFP-Snc1-PEM structures, % cells with GFP-Snc1-PEM outside vacuole, and % cells with GFP-Snc1-PEM inside the vacuole. +/- and error bars represent STDEV. Results in this figure represent at least two independent experiments.

The macro-ER-phagy phenotype of the single *ypt1-1* mutation is more severe than those of a single deletion of any Atg. Importantly, *atg9∆* suppresses the ER-phagy phenotype of *ypt1-1*. First, the level of GFP-Snc1-PEM in *ypt1-1* mutant cells is ~20-fold higher than its level in WT cells. In *ypt1-1 atg9∆* double mutant cells, the level is reduced to ~5.5 fold (Fig 3A, left). Second, whereas 85% of *ypt1-1* mutant cells contain large intracellular GFP-Snc1-PEM structures, only 28% of the *ypt1-1 atg9∆* double mutant cells contain small structures, similar to *atg9∆* mutant cells (Fig 3B, left). Third, the UPR is also lower in the *ypt1-1 atg9∆* double mutant then in the single *ypt1-1* mutant cells (Fig 3C, left).

Together, these results show that Atg9 functions upstream of Ypt1 and core Atgs in macro-ER-phagy. The fact that GFP-Snc1-PEM does not accumulate in large aberrant structures in *atg9∆* mutant cells as it does in the other mutant cells, suggests that Atg9 plays a role in exit of macro-ER-phagy cargo from the ER. Interestingly, UPR induction seems to be dependent on the assembly of these structures.

One prediction from these results is that Atg9 would accumulate on aberrant ER structures in *ypt1-1* mutant cells. To determine whether this is the case, WT and *ypt1-1* cells expressing endogenously tagged Atg9-mCherry and overexpressing GFP-Snc1-PEM were analyzed by live-cell microscopy. Whereas in WT cells the Atg9 puncta do not co-localize with the PM-localized GFP-Snc1-PEM, in *ypt1-1* mutant cells Atg9 co-localizes with the aberrant GFP-Snc1-PEM structures (Fig 3D). Thus, Atg9 is required for the formation of the aberrant ER structures in *ypt1-1* mutant cells and is present on their membrane.

### Characterization of macro-ER-phagy

To better characterize macro-ER-phagy, we determined whether it is affected by mutations in Sec12 and Vps4, which mediate the exocytic pathway and autophagy-independent delivery to the lysosome, respectively. Sec12 mediates ER-to-Golgi transport [37], which is also regulated by Ypt1 [38]. Because in addition to their ER-to-Golgi block, *sec12ts* mutant cells are also defective in ERAD at their restrictive temperature [39], it was expected that they would accumulate some GFP-Snc1-PEM in their ER even at permissive temperature. Indeed, the level of GFP-Snc1-PEM is increased ~3.5 fold when compared to WT cells, 40–50% of the cells accumulate some of it in their ER, and UPR is induced (S3 Fig). However, there are three main differences between the accumulation of GFP-Snc1-PEM in *sec12-ts* and *ypt1-1* mutant cells. First, whereas overexpression of GFP-Snc1-PEM in *ypt1-1* mutant cells results in a two-fold increase of the UPR, UPR is higher in *sec12ts* mutant cells that do not overexpress GFP-Snc1-PEM (S3C Fig). This result suggests that UPR induction in *sec12ts* mutant cells is due to a defect in ER exit of multiple proteins, and triggering the macro-ER-phagy pathway by excess GFP-Snc1-PEM might partially relieve the ER stress in these mutant cells. Second, while deletion of *ATG9* masks the phenotypes of *ypt1-1* (e.g., GFP-Snc1-PEM structures accumulation and UPR induction), it exacerbates those of *sec12ts* mutation (Fig 3A–3C, right). This result indicates that Atg9 and Sec12 do not function in the same pathway. Most importantly, we have previously shown that *ypt1-1* mutant cells, regardless if they are defective in vacuolar proteolysis or not, accumulate GFP-Snc1-PEM outside their vacuole [22]. In contrast, whereas there is no GFP-Snc1-PEM in vacuoles of *sec12ts* mutant cells, it does accumulate in the proteolysis-defective vacuoles of *sec12ts pep4∆* double-mutant cells (Fig 3E). Because GFP-Snc1-PEM can get to the vacuole only through autophagy and not through endocytosis [27], *sec12ts* mutant cells are not defective in autophagy. Together, these results show that unlike Ypt1, Sec12 is not required for macro-ER-phagy.

Vps4 is required for transport to the lysosome from the PM or the Golgi through late endosomes [40]. To confirm that GFP-Snc1-PEM reaches the vacuole from the ER and not from the Golgi, macro-ER-phagy of overexpressed GFP-Snc1-PEM was determined in *vps4∆* mutant cells. In all three aforementioned assays, *vps4∆* mutant cells behave like WT cells (S4A–S4C Fig).

Recently, a micro-ER-phagy process that does not require known autophagy machinery was described [15]. This process can be induced by adding DTT or tunicamycin to cells and observed by the presence of ER membrane “whorls” in vacuole of proteolysis-defective mutants. To confirm that the Ypt1 is not required for micro-ER-phagy, DTT-dependent formation of ER-membrane whorls was compared in *YPT1* and *ypt1-1* mutant cells defective in vacuolar proteolysis (*pep4∆ prb1∆*). Accumulation of ER whorls was similar in both strains (S4D Fig), indicating that Ypt1 does not play a role in micro-ER-phagy. Therefore, GFP-Snc1-PEM accumulation in *ypt1-1* mutant cells is caused by a defect in a process distinct from micro-ER-phagy.

**Fig 4:**
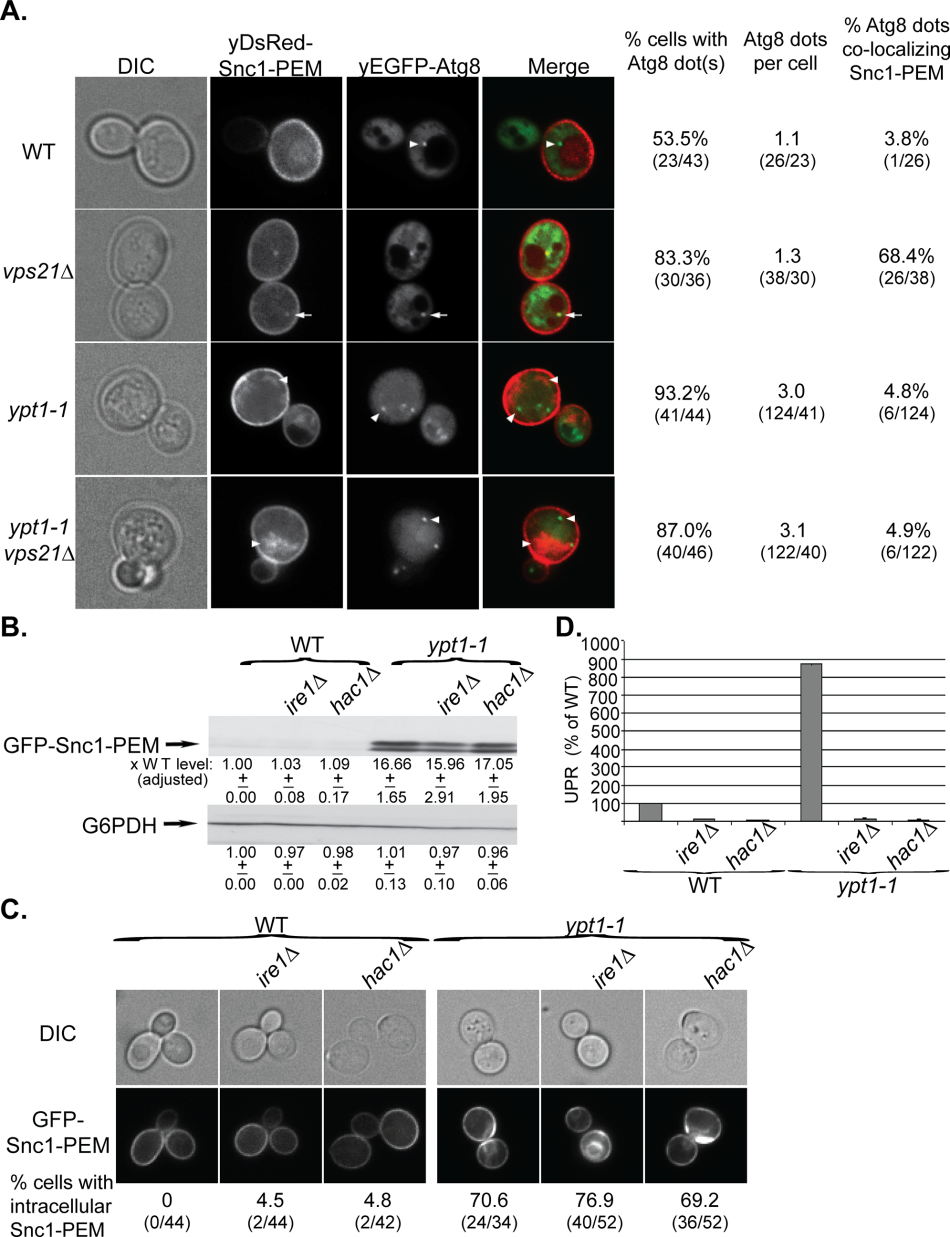
GFP-Snc1-PEM accumulates in APs of *vps21∆* mutant cells and macro-ER-phagy is independent of UPR induction. **A.** The *ypt1-1* mutation is epistatic to *vps21∆* in ER-phagy. yDsRed-Snc1-PEM was overexpressed in WT, *vps21∆*, *ypt1-1* and *ypt1-1 vps21∆* double mutant cells that also expressed the autophagosomal marker yEGFP-Atg8. Cells were analyzed by live-cell microscopy. Shown from left to right: DIC, DsRed, GFP, merge, % cells Atg8 dots, number of Atg8 dots per cell, and % cells in which the Atg8 dots co-localize with Snc1-PEM. About 50% of WT and 85% of *vps21∆* mutant cells contain ~1 dot of Atg8 representing the AP. Importantly, in ~70% of the *vps21∆* mutant cells Snc1-PEM co-localizes with the APs, as compared to ~4% in WT cells. In contrast, ~90% *ypt1-1* and *ypt1-1 vps21∆* mutant cells contain three APs per cell, and Snc1-PEM does not co-localize with them. Arrows point to co-localization; arrowheads point to either Atg8 dots or GFP-Snc1-PEM that do not co-localize. **B-D.** UPR induction is not required for macro-ER-phagy. The UPR regulators Ire1 or Hac1 were deleted in the WT and *ypt1-1* mutant cells. The following effects of overexpression of GFP-Snc1-PEM in WT and *ypt1-1* mutant cells, without and with *ire1∆* or *hac1∆*, were analyzed as described for Fig 1A–1C, respectively: protein level (**B**), accumulation of GFP-Snc1-PEM in aberrant structures (**C**), and UPR induction (**D**). **B.** Deletion of either Ire1 or Hac1 does not affect the level of Snc1-PEM accumulation in WT or *ypt1-1* mutant cells. **C.** Deletion of either Ire1 or Hac1 does not affect the percent of WT and *ypt1-1* mutant cells that accumulate aberrant intra-cellular Snc1-PEM. Shown from top to bottom: DIC, GFP, and % cells with intracellular Snc1-PEM structures. **D.** Deletion of either Ire1 or Hac1 obliterate UPR in both WT and *ypt1-1* mutant cells. +/- and error bars represent STDEV. Results in this figure represent at least two independent experiments.

Our finding that the core autophagy machinery is required for delivery of GFP-Snc1-PEM to the vacuole supports the idea that the process described here is macro-ER-phagy. To confirm this idea, we determined whether yDsRed-Snc1-PEM passes through APs en route to the vacuole. Recently, we showed that the Rab5 homolog Vps21 plays a role in autophagy as *vps21∆* mutant cells accumulate AP structures marked by Atg8 under starvation [24]. This indicates that autophagy is blocked in *vps21∆* mutant cells after the formation of APs. Under normal growth conditions, both WT and *vps21∆* mutant cells have one Atg8 dot per cell, representing the phagophore or AP [41]. Co-localization of overexpressed yDsRed-Snc1-PEM with yEGFP-Atg8 was observed in ~70% of *vps21∆* mutant cells, as compared to ~4% of WT cells (Fig 4A, top). This *vps21∆* phenotype is also different from that of the *ypt1-1* single mutation and the *ypt1-1 vps21∆* double mutation. As we have previously shown, *ypt1-1* mutant cells are defective in PAS formation and contain multiple Atg8 dots per cell [22,42]. Even though yDsRed-Snc1-PEM accumulates in *ypt1-1* single- and *ypt1-1 vps21∆* double-mutant cells, it does not co-localize with the multiple Atg8 dots (Fig 4A, bottom). The finding that the *ypt1-1* mutation overrides the *vps21∆* phenotype indicates that Ypt1 and Vps21 function sequentially in the same pathway. Moreover, the co-localization of yDsRed-Snc1-PEM with Atg8 in *vps21∆* mutant cells indicates that it passes through APs en route to the vacuole and confirms that this pathway is macro-ER-phagy.

UPR plays a role in ERAD [5] and is induced when macro-ER-phagy is blocked [17]. To determine whether UPR is required for macro-ER-phagy, accumulation of overexpressed GFP-Snc1-PEM was tested in *ire1∆* and *hac1∆* mutant cells, which are defective in UPR induction. In WT (*YPT1*) cells, deletion of either *IRE1* or *HAC1* abolishes UPR, but does not result in accumulation of GFP-Snc1-PEM (Fig 4B–4D, left). In *ypt1-1* mutant cells, deletion of either *IRE1* or *HAC1* abolishes UPR induction, but does not affect accumulation of GFP-Snc1-PEM (Fig 4B–4D, right). These results show that Ire1/Hac1-dependent UPR induction is not required for macro-ER-phagy and does not affect the formation of aberrant ER structures in *ypt1-1* mutant cells.

### Macro-ER-phagy cargos

**Fig 5:**
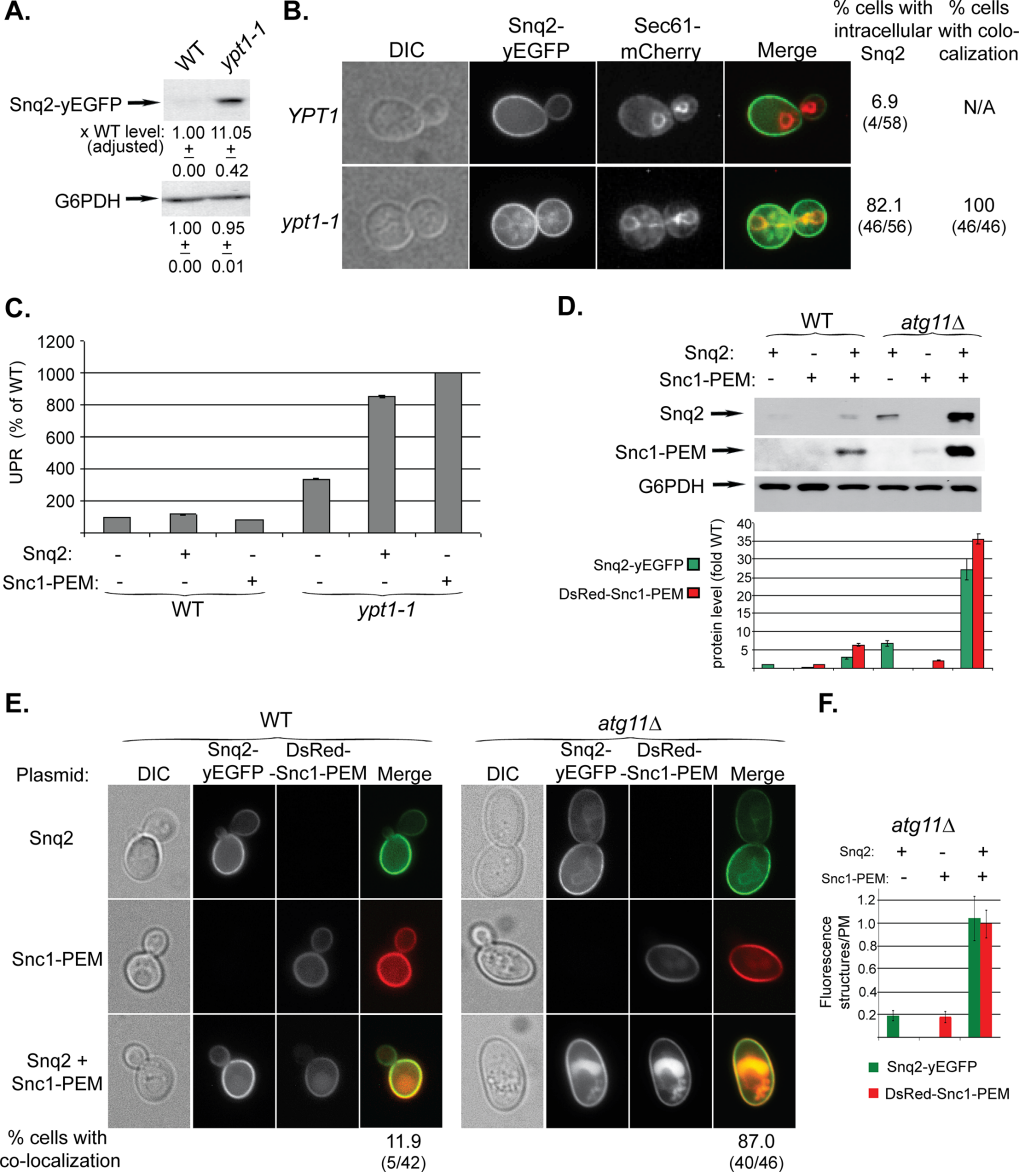
Snq2-yEGFP is another macro-ER-phagy cargo and its co-overexpression with DsRed-Snc1-PEM has a synergistic effect. **A-C.** Overexpressed Snq2-GFP accumulates in the ER and induces UPR in *ypt1-1* mutant cells. Snq2-yEGFP was overexpressed in WT and *ypt1-1* mutant cells and the following effects were analyzed as described for Fig 1A–1C, respectively: increase in the level of Snq2-yEGFP protein (**A**), accumulation of Snq2-yEGFP in aberrant ER structures (**B**), and induction of the UPR response (**C,** GFP-Snc1-PEM is shown for comparison). **B.** The ER marker Sec61 was tagged with mCherry at its C-terminus in WT and *ypt1-1* mutant cells. Shown from left to right: DIC, GFP, mCherry, Merge, % cells with intracellular Snq2 and % cells in which Snq2 co-localized with Sec61. **D-F.** Synergistic effect of co-overexpression of Snq2-GFP with DsRed-Snc1-PEM in *atg11∆* mutant cells. WT (left) and *atg11∆* mutant cells (right) were transformed with plasmids for overexpression of Snq2, Snc1-PEM or both Snq2 and Snc1-PEM. **D.** Immunoblot analysis and quantification. Shown from top to bottom: Snq2-yEGFP (using anti-GFP antibodies), Ds-Red-Snc1-PEM (using anti-Snc1 antibodies), G6PDH (loading control), and a bar graph showing fold increase of Snq2-yEGFP (green) and Ds-Red-Snc1-PEM (red) in *atg11∆* mutant cells over WT. **E.** Accumulation of macro-ER-phagy cargos in aberrant intracellular structures using live-cells microscopy. Shown from left to right: DIC, GFP, DsRed and Merge. Shown from top to bottom: Snq2, Snc1-PEM, Snq2+Snc1-PEM, and % cells with co-localization (relevant only for co-overexpression) (% cells with ER-phagy cargo accumulation in aberrant intracellular structures is shown in S5A Fig). **F.** Fluorescence level of intracellular structures (ratio over PM) in *atg11∆* mutant cells. The bar graph shows Snq2-yEGFP (green) and Ds-Red-Snc1-PEM (red) fluorescence (20 cells were analyzed for each strain). When co-overexpressed in *atg11∆* mutant cells, the fluorescence level of either protein accumulating in aberrant structures is ~5 fold higher than when overexpressed individually. +/- and error bars represent STDEV. Results in this figure represent at least two independent experiments.

We have shown that overexpressed GFP-Snc1-PEM, a chimeric integral PM protein, can serve as a cargo for macro-ER-phagy [17]. We wished to identify a native protein that when overexpressed would be a cargo for ER-phagy. For this purpose we chose the PM multi-drug transporter Snq2 that contains multiple trans-membrane domains. Snq2 tagged at its C-terminus with yEGFP was overexpressed from a 2μ plasmid in WT and *ypt1-1* mutant cells. In *ypt1-1* mutant cells, like GFP-Snc1-PEM, the level of Snq2-yEGFP was increased (by ~10-fold), it accumulated in aberrant ER structures in 80% of the cells, and UPR was induced (Fig 5A–5C). Thus, overexpressed Snq2-yEGFP, like GFP-Snc1-PEM, is transported through macro-ER-phagy for degradation in the vacuole.

To study the effect of overexpression of multiple membrane proteins on macro-ER-phagy, Snq2-yEGFP and DsRed-Snc1-PEM were co-overexpressed in WT and *atg11∆* mutant cells. In WT cells, while overexpression of a single membrane protein did not have any effect, overexpression of two proteins resulted in some increase in the protein levels, and they co-localized in the vacuole of ~12% of the cells (Figs 5D and 5E and S5A). In *atg11∆* mutant cells, while overexpression of a single membrane protein had a moderate phenotype, overexpression of the two resulted in a synergistic effect. The protein levels were increased >25 fold over that of WT cells (single protein levels were increased 2.5–6 fold) (Fig 5D). In 87% of the cells the two proteins co-localized in aberrant intra-cellular structures (compared to 50–65% for single protein) (Figs 5E and S5A). Moreover, the fluorescence of these intra-cellular structures was 5-fold brighter than that of each single protein (Fig 5F). Finally, whereas overexpression of a single membrane protein did not affect the growth of *atg11∆* mutant cells, overexpression of two caused a growth defect (S5B Fig). These results suggest that when macro-ER-phagy is partially defective, as in *atg11∆* mutant cells, the extra burden of overexpression of multiple membrane proteins can be detrimental.

Together, these results indicate that excess of integral membrane proteins, such as Snc1-PEM and Snq2, are transported to the vacuole through macro-ER-phagy.

### ER-resident proteins in macro-ER-phagy

**Fig 6:**
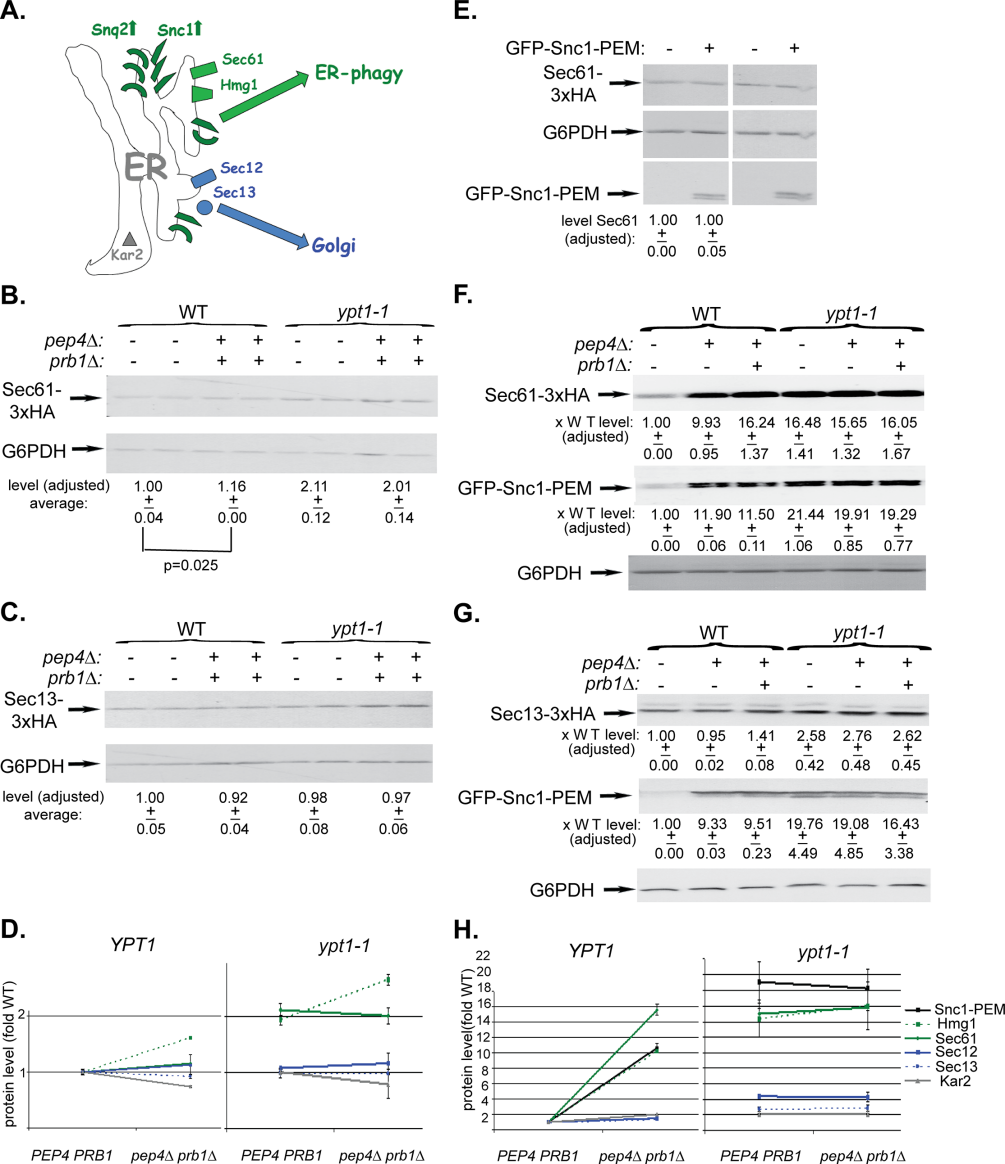
Immunoblot analysis of ER-resident proteins with and without overexpression of GFP-Snc1-PEM. **A.** A diagram showing two groups of ER-resident proteins: those that become macro-ER-phagy cargos (in green), and those that do not (in blue or grey). Like overexpressed Snc1-PEM or Snq2, the ER-resident membrane proteins Sec61 (translocon subunit) and Hmg1 (sterol biogenesis) are transported to the vacuole via macro-ER-phagy. In contrast, the ER-to-Golgi exit regulators Sec12 and Sec13 and the ER lumen chaperone Kar2 are not co-transported to the lysosome through macro-ER-phagy. Four or six different strains were used for this analysis: WT (*YPT1*), *pep4∆*, and *pep4 prb1∆*, *ypt1-1*, *ypt1-1 pep4∆*, and *ypt1-1 pep4∆ prb1∆*. In each strain, one ER-resident protein was tagged at its C-terminus (except for Kar2). (The *pep4∆* strains were not used in all experiments because they do not show the full defect). The level of ER-resident proteins was determined by immunoblot analysis in cells that either do not (**B-D**) or do overexpress GFP-Snc1-PEM (**F-H**). **B-C.** Sec61-3xHA (B) and Sec13-3xHA (C) expressing cells (2 independent un-transformed colonies) were tested by immunoblot analysis (using anti-HA antibodies). Shown from top to bottom: strain genotype, HA-tagged protein, G6PDH (loading control), quantification of HA-tagged protein expressed as average fold of WT (p-value = 0.025 for Sec61). **D.** Summary of immunoblot analyses of ER-resident proteins level in cells that do not overexpress GFP-Snc1-PEM: WT (*PEP4 PRB1*) vs *pep4∆ prb1∆* (left), *ypt1-1 PEP4 PRB1* vs *ypt1-1 pep4∆ prb1∆* (right). Immunoblots and quantification are shown in Fig 6B–6C and S6A–S6C Fig. In *YPT1* cells, the levels of Sec61 and Hmg1 (green) are slightly increased (14 and 38%, respectively) in strains defective in vacuolar proteolysis; the levels of Sec12, Sec13 and Kar2 are not increased (blue and grey). The levels of Sec61 and Hmg1 (green) are increased by 2-2.5 fold in *ypt1-1* mutant cells when compared to WT cells, regardless if they are defective in vacuolar proteolysis or not. In contrast, the levels of Sec12, Sec13 and Kar2 are only slightly increased (blue and grey). **E.** The level of the ER-resident protein Sec61 is similar whether GFP-Snc1-PEM is overexpressed or not. Wild-type cells were transformed with a 2μ plasmid, either empty or for overexpression of GFP-Snc1-GEM (2 independent transformants). Shown from top to bottom: plasmid, Sec61, G6PDH (loading control), GFP-Snc1-PEM, and quantification of Sec61 expressed as average fold of WT with empty plasmid. **F-G.** Sec61-3xHA (F) and Sec13-3xHA (G) expressing cells were transformed with a 2μ plasmid for overexpression of GFP-Snc1-PEM. Cell lysates were tested by immunoblot analysis (using ant-HA and anti-GFP antibodies). Shown from top to bottom: strain genotype, the specific ER-resident protein tested, quantification of the ER-resident protein bands expressed as average fold of WT, GFP-Snc1-PEM, quantification of the GFP-Snc1-PEM bands expressed as average fold of WT, and G6PDH (loading control). **H.** Summary of immunoblot analysis of ER-resident proteins level in cells overexpressing GFP-Snc1-PEM: WT (*PEP4 PRB1*) vs *pep4∆ prb1∆* (left), *ypt1-1 PEP4 PRB1* vs *ypt1-1 pep4∆ prb1∆* (right). Immunoblots and quantification are shown in Fig 6F and 6G and S6F–S6H Fig. In *YPT1* cells, like Snc1-PEM (black), the levels of Hmg1 and Sec61 (green) are increased >10 fold in strains defective in vacuolar proteolysis. In contrast, the levels of Sec12, Sec13 and Kar2 are not changed (blue and grey). Like Snc1-PEM (black), the protein levels of Hmg1 and Sec61 (green) are increased ~15 fold in *ypt1-1* mutant cells when compared to WT cells, regardless if they are defective in vacuolar proteolysis or not. In contrast, the levels of Sec12, Sec13 and Kar2 are only slightly increased (blue and grey). +/- and error bars represent STDEV. Results in this figure represent at least two independent experiments.

We wished to determine which ER-resident proteins are transported together with GFP-Snc1-PEM to the vacuole via the macro-ER-phagy pathway. Five ER-resident proteins were used for this analysis: three integral membrane proteins, Sec61 (translocon subunit), Hmg1 (sterol biogenesis), and Sec12 (ER-exit sites), a cytoplasmic coat protein, Sec13 (ER exit sites), and an ER-lumen chaperon, Kar2 (BiP) (Fig 6A). Protein accumulation was determined by immunoblot and microscopy analyses in WT (*YPT1*) and *ypt1-1* mutant cells in which vacuolar proteolysis was normal (*PEP4 PRB1*) or defective (*pep4∆ prb1∆*). The analysis was done without and with over-expression of GFP-Snc1-PEM.

We have previously shown that some resident-ER proteins accumulate in *ypt1-1* mutant cells when compared to WT cells even without overexpression of GFP-Snc1-PEM. This phenotype was observed using live-cell microscopy and tagged resident ER proteins, e.g., Hmg1 and Sec61 [17]. Here, the microscopy analysis is supported by an immunoblot analysis. The levels of Sec61 and Hmg1 were determined in cells that do not overexpress GFP-Snc1-PEM. In *YPT1* (WT) cells defective in vacuolar proteolysis (*pep4∆ prb1∆*), the levels of Sec61 and Hmg1 were increased by 1.2–1.6 fold. In *ypt1-1*, regardless if the vacuolar proteolysis is normal or defective, the levels of Sec61 and Hmg1 were increased by ~2–2.6 fold (Figs 6B and 6D and S6A). In contrast, the levels of Sec12, Sec13 and Kar2 were not increased in these mutant cells (Figs 6C and 6D and S6B and S6C). Therefore, ~20–40% of some ER resident proteins, but not all, are shuttled to the vacuole for degradation in WT and *ypt1-1* mutant cells even in cells that do not over-express GFP-Snc1-PEM.

The previously observed Hmg1 and Sec61 accumulation in *ypt1-1* mutant cells was exacerbated when GFP-Snc1-PEM was overexpressed. In addition, we have previously shown that in *YPT1* (WT) cells GFP-Snc1-PEM is transported to the vacuole for degradation [17]. We wished to determine whether ER-resident proteins are shuttled to the vacuole for degradation with over-expressed GFP-Snc1-PEM.

Hmg1 and Sec61 behaved like Snc1-PEM in wild type cells that overexpress GFP-Snc1-PEM. Immuno-blot analysis shows that their levels increased by 10 fold in cells defective in vacuolar proteolysis, indicating that they were delivered to the vacuole (Figs 6F and 6H and S6F). The degradation rate of Sec61 was compared in WT cells, which either over-express GFP-Snc1-PEM or not, using cycloheximide to inhibit protein translation. The half-life of Sec61 is shortened by 7 fold in cells over-expressing GFP-Snc1-PEM (from 29 to 4 hours; S6D Fig). The finding that the stability of the ER resident protein Sec61 is reduced in WT cells over-expressing GFP-Snc1-PEM further supports the idea that it is shuttled to the vacuole for degradation under these conditions. However, in spite of the fact that ~95% of Sec61 and Hmg1 were degraded in cells overexpressing GFP-Snc1-PEM, their steady-state level did not change (Figs 6E and S6E). In contrast to Sec61 and Hmg1, the levels of Sec12, Sec13 and Kar2 in vacuolar proteolysis defective cells overexpressing GFP-Snc1-PEM were very slightly changed (1.5–2 fold) (Figs 6G and 6H and S6G–S6H, left). Thus, in WT cells, >90% of some, but not all, ER resident proteins are shuttled for degradation in the vacuole with overexpressed GFP-Snc1-PEM.

In *ypt1-1* mutant cells that overexpress GFP-Snc1-PEM, the levels of Hmg1 and Sec61, like that of Snc1-PEM, were increased by ~15 fold regardless whether vacuolar proteolysis was normal or defective, indicating that they accumulate before reaching the vacuole. In contrast, the levels Sec12, Sec13 and Kar2 increased by 4, 3, and 2 fold, respectively (Figs 6F–6H and S6F–S6H, right).

**Fig 7:**
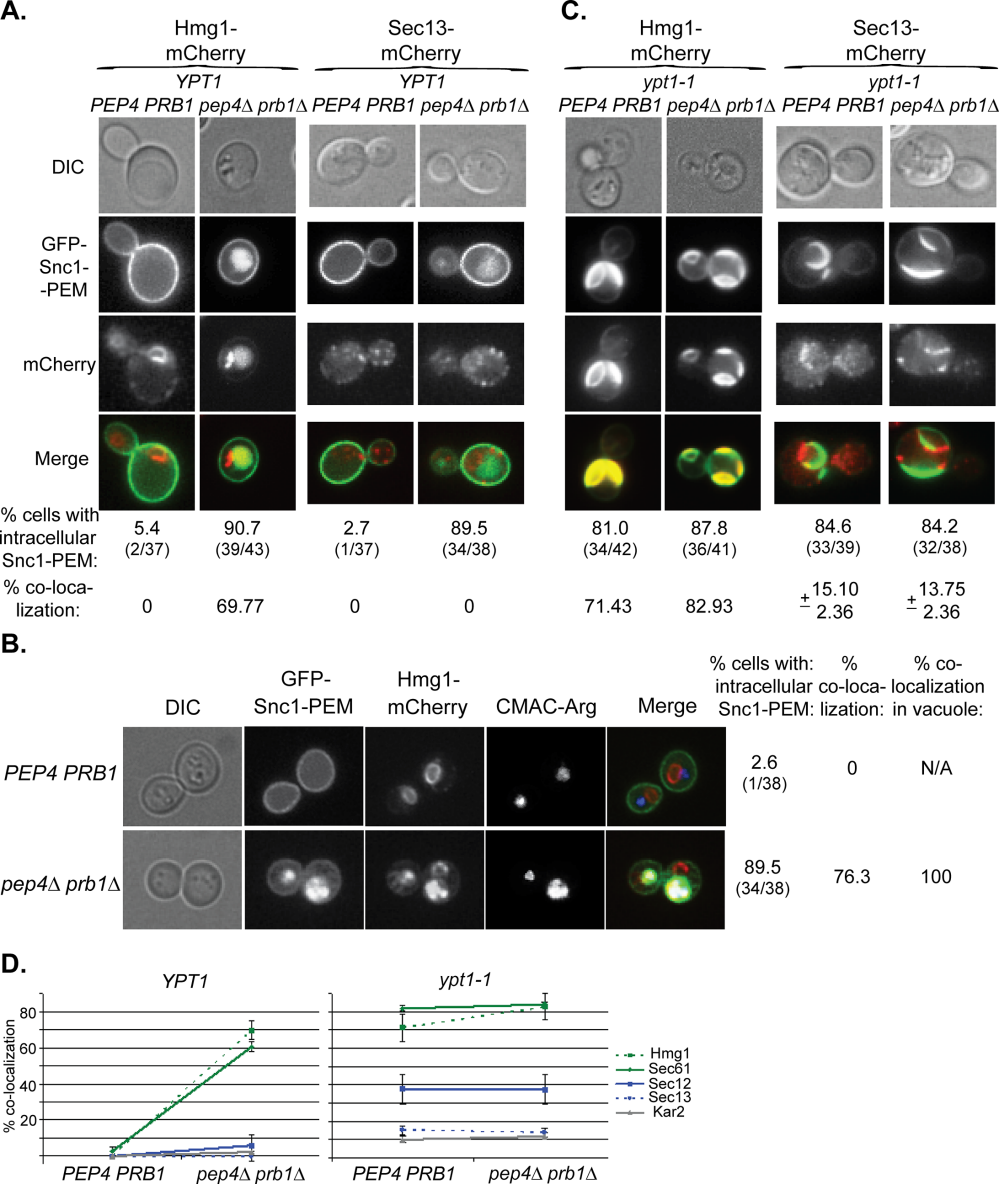
Live-cell microscopy analysis of ER-resident proteins upon overexpression of GFP-Snc1-PEM. **A.** In cells defective in vacuolar proteolysis (*pep4∆ prb1∆*), Hmg1 co-localizes with 70% of intra-cellular Snc1-PEM, but Sec13 does not (at least 36 cells were analyzed for each strain). No co-localization was observed for resident ER proteins and overexpressed Snc1-PEM in WT cells (*PEP4 PRB1*). Hmg1 (left) and Sec13 (right) were tagged with mCherry at their C-terminus in *PEP4 PRB1* (WT) and *pepb4∆ prb1∆* mutant cells. Cells were transformed with a 2μ plasmid for overexpression of GFP-Snc1-PEM and analyzed by live-cell microscopy. Shown from top to bottom: DIC, GFP, mCherry, merge (yellow), % cells with intra-cellular Snc1-PEM, and % co-localization of the ER protein with intra-cellular Snc1-PEM. **B.** The ER-resident protein Hmg1 is delivered to the vacuole with GFP-Snc1-PEM. The vacuole of *PEP4 PRB1* (WT) and *pepb4∆ prb1∆* mutant cells expressing Hmg1-mCherry and overexpressing GFP-Snc1-PEM (see Panel A, left) was stained with CMAC-Arg (blue), and the cells were analyzed by live-cell microscopy. In WT cells (top), there is no intracellular Snc1-PEM and the red-labeled ER is distinguished from the blue vacuole. In contrast, in ~75% *pep4∆ prb1∆* mutant cells (bottom), which are defective in vacuolar proteolysis, Hmg1 (red) co-localizes with Snc1-PEM (green) in the vacuole (blue). Shown from left to right: DIC, GFP, mCherry, blue, merge (white shows merge of the three colors), % cells with intracellular Snc1-PEM, % cells in which Hmg1 with Snc1-PEM co-localize, and % co-localization of the two proteins with the vacuolar dye (38 cells were analyzed for each strain). **C.** >80% of *ypt1-1* mutant cells, regardless if they are *PEP4 PRB1* or *pep4∆ prb1∆*, accumulate aberrant GFP-Snc1-PEM structures. Hmg1 co-localizes with >70% of these structures, whereas Sec13 co-localizes with only ~15% of these structures (36–43 cells were analyzed for each strain). The experiment was done as described for Panel A. **D.** Summary of microscopy analysis of ER-resident proteins co-localization with GFP-Snc1-PEM in cells overexpressing GFP-Snc1-PEM: WT (*PEP4 PRB1*) vs *pep4∆ prb1∆* (left), and *ypt1-1 PEP4 PRB1* vs *ypt1-1 pep4∆ prb1∆* (right). Microscopy and quantification are shown in Fig 7A and 7C and S7 Fig. In *YPT1* cells, whereas >60% of Hmg1 and Sec61 (green) co-localize with Snc1-PEM in strains defective in vacuolar proteolysis, Sec12, Sec13 and Kar2 are not changed significantly (blue and grey). Hmg1 and Sec61 (green) co-localize with Snc1-PEM in >70% of *ypt1-1* and >80% of *ypt1-1 pep4∆ prb1∆* mutant cells, whereas Sec13 and Kar2 (blue and grey) do so in <15% of the cells. The level of Sec12 co-localization with Snc1-PEM is intermediate, ~38% in *ypt1-1* and *ypt1-1 pep4∆ prb1∆*. Error bars represent STDEV. Results in panels A and B represent at least two independent experiments.

In the microscopy analysis, Hmg1 and Sec61 also behaved like GFP-Snc1-PEM. In *YPT1* (WT) cells defective in vacuolar proteolysis, 60–70% of the intra-cellular GFP-Snc1-PEM co-localized with mCherry-tagged Hmg1 and Sec61 (Figs 7A and 7D and S7A). The proteins probably co-localize in the vacuole because co-localization is observed only in cells defective in vacuolar proteolysis and, using a vacuolar membrane stain, we have previously shown that GFP-Snc1-PEM accumulates inside the vacuole of *pep4∆* cells [17]. This point was confirmed for Hmg1, which co-localizes with Snc1-PEM in the vacuole, using three-color fluorescence microscopy (Fig 7B). In contrast, very little co-localization was observed for Sec13, Sec12 and Kar2 (Figs 7A and 7D and S7A). In *ypt1-1* mutant cells, >70% of GFP-Snc1-PEM co-localized with Hmg1 and Sec61, regardless of the vacuolar proteolysis state. For Sec12, Sec13 and Kar2, the levels were lower: 38, 14 and 12%, respectively (Figs 7C and 7D and S7B).

These results show that ~20–40% of some, but not all, ER-resident proteins are transported to the vacuole through macro-ER-phagy in WT cells. In cells over-expressing excess integral membrane proteins such as Snc1-PEM, >90% of these ER proteins are corralled to this pathway. Interestingly, even though the ER resident protein Sec61 is shuttled to the vacuole for the degradation when GFP-Snc1-PEM is overexpressed, its steady state level does not change.

## Discussion

**Fig 8:**
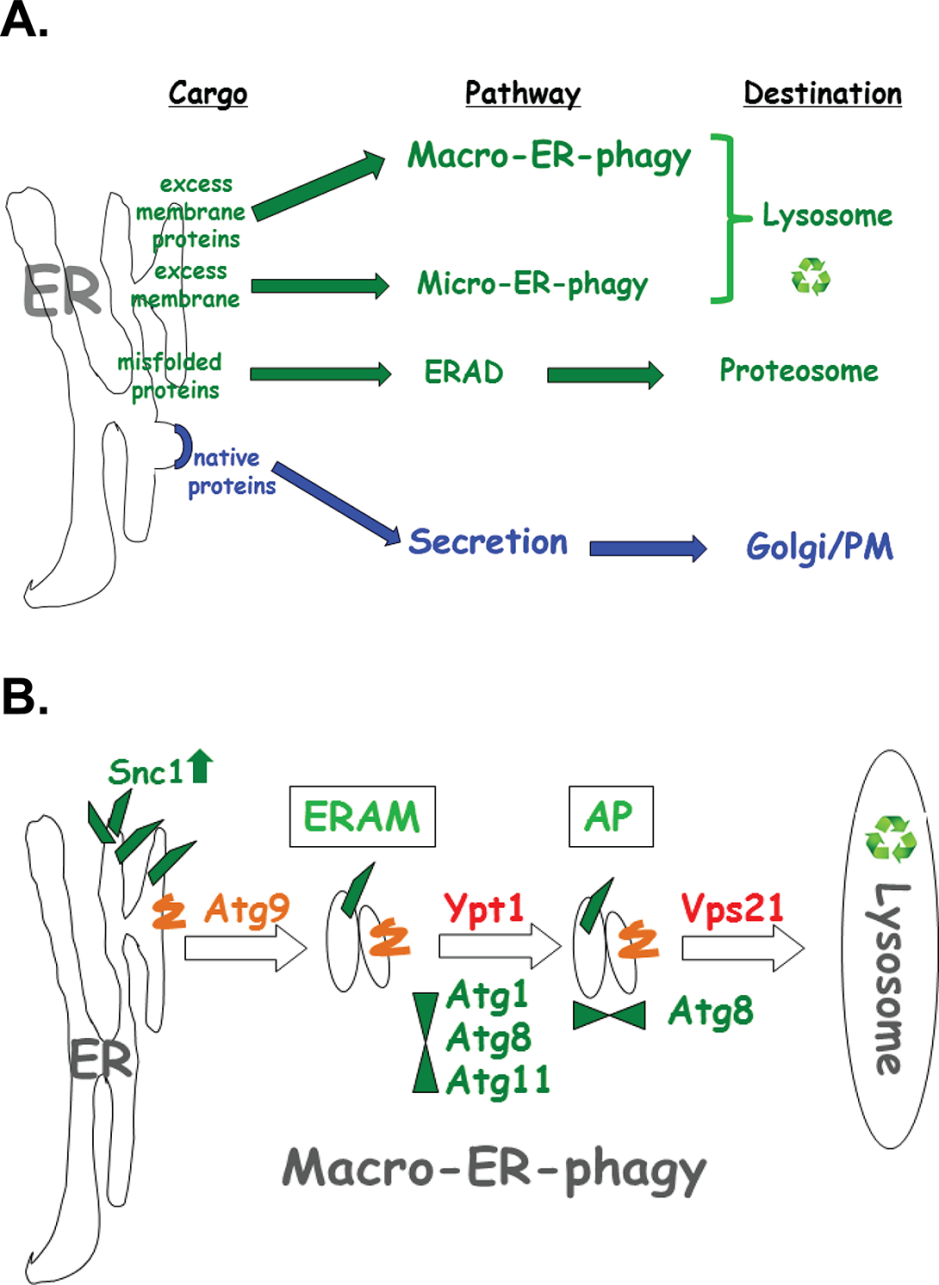
The macro-ER-phagy pathway and ERQC. **A.** Diagram illustrating three major ERQC pathways. Whereas native proteins are transported from the ER to through the Golgi for secretion (blue), three different pathways shuttle membrane and proteins for degradation by (green). ERAD shuttles misfolded proteins to the proteasome for degradation. Upon ER stress induced by tunicamycin or DTT, excess membrane is shuttled to the lysosome via micro-ER-phagy, which does not require neither Atgs nor Ypt GTPases. Here, macro-ER-phagy, which requires Atgs and Ypt GTPases, is shown to shuttle excess membrane proteins for degradation in the lysosome. **B.** Model of three discreet steps in macro-ER-phagy that carry ER-phagy cargo (e.g., overexpressed GFP-Snc1-PEM) to the lysosome for degradation: 1) Atg9-dependent formation of ER-to-autophagy membranes (ERAM) and induction of UPR; 2) Ypt1-dependent assembly of ERAM with PAS proteins (e.g, Atg1, Atg8 and Atg11); UPR is induced in mutant cells blocked in this step; 3) Vps21-dependent delivery of APs carrying ER-phagy cargo to the lysosome. See text for discussion.

Results presented here define a novel ER quality-control pathway, macro-ER-phagy. This pathway delivers excess of integral-membrane proteins from the ER to the lysosome for degradation and requires core autophagy machinery and at least two Ypt GTPases, Ypt1 and Vps21 (Ypt51). It was defined as macro-ER-phagy based on the requirement of core Atgs and the accumulation of the cargo in Atg8-marked autophagic structures (phagophore and APs) in *vps21∆* mutant cells. Macro-ER-phagy is different from nonselective autophagy because it does not require starvation or prolonged ER stress, and from other specific autophagy processes, because it does not require known specific Atgs (like Atg19, Atg32, and Atg36). It is also different from micro-ER-phagy, which does not require Atgs [15] or Ypt1 GTPase (shown here). Finally, macro-ER-phagy is different from the other ERQC pathway ERAD in which proteins are extracted from the ER lumen through the membrane and delivered to the proteasome for degradation (Fig 8A). When macro-ER-phagy is impaired, UPR is induced, indicating that accumulation of its cargo causes ER stress. However, UPR induction is not required for macro-ER-phagy.

### Macro-ER-phagy cargo

The cargo shuttled through the macro-ER-phagy pathways is different from cargo delivered by other known ERQC pathways. Whereas the cargo of the ERAD and micro-ER-phagy pathway are misfolded proteins (recognized in the ER lumen or in the ER membrane) and extra membrane, respectively [3,15], the cargo of the macro-ER-phagy is excess of integral-membrane proteins. Based on the fact that one such cargo, GFP-Snc1-PEM, does not have a lumenal domain, we propose that the macro-ER-phagy cargo is recognized in the cytoplasm.

What fraction of integral-membrane proteins is transported through basal macro-ER-phagy? Without overexpression of a membrane protein, about 20–50% of the ER resident proteins Sec61 and Hmg1 are transported to the vacuole for degradation. This estimate is based on a 1.2–1.6 fold increase in cells defective in vacuolar proteolysis, and on the 2–2.5 fold accumulation in *ypt1-1* mutant cells regardless of whether vacuolar proteolysis is normal or not. This estimate is within the 30–75% range previously reported for ER proteins that do not pass the ERQC [1].

Importantly, cells that overexpress the integral-membrane protein GFP-Snc1-PEM, shuttle ~95% of this protein to the vacuole for degradation via macro-ER-phagy. This fraction is estimated based on the following two results: ~20-fold increase in GFP-Snc1-PEM level in cells defective in macro-ER-phagy (e.g., *ypt1-1*), and >10-fold increase in cells defective in vacuolar proteolysis (*pep4∆ prb1∆*). Moreover, >90% of some ER-resident membrane proteins (e.g., Sec61 and Hmg1) are also transported through macro-ER-phagy for degradation in cells overexpressing GFP-Snc1-PEM. In spite of this increase in the degradation of ER resident proteins upon over-expression of a single integral membrane protein, the steady state level of Sec61 and Hmg1 remained unchanged.

We propose that macro-ER-phagy is a “housekeeping” pathway that delivers excess resident-ER membrane proteins to the lysosome for degradation. When an integral membrane protein is overexpressed, ~95% of this protein is shuttled through this pathway together with >90% of the ER that contain certain resident-ER membrane proteins. Therefore, macro-ER-phagy of some resident ER proteins is induced from 20–50% to >90% by over-expression of a single membrane protein. The observation that the steady state level did not plunge under these conditions suggests that cells regulate the level of these ER resident proteins.

### Delineation of three distinct steps of the macro-ER-phagy pathway

Based on data presented here, we delineate three sequential steps of the macro-ER-phagy pathway: In the first Atg9-dependent step, membranes containing macro-ER-phagy cargo form ER-to-autophagy membranes (ERAM). If this step is blocked, cargo accumulates in the ER, but UPR is not induced. In the second step, which is dependent on Ypt1, ERAM and the core Atgs form the PAS. If this step is blocked, ERAM accumulate and UPR is induced, even though UPR induction is not required for macro-ER-phagy. In the third Atg8- and Vps21-mediated step, APs are formed and fuse with the lysosome (see model in Fig 8B).

Atg9, the only integral-membrane core Atg, was implicated in delivering membrane to PAS [7,8]. However, until now its function could not be separated from that of the other PAS organizers. Because the macro-ER-phagy phenotype of *atg9∆* is different from, and can mask, the phenotypes of *ypt1-1*, *atg1∆* and *atg11∆*, the two steps could be separated and their order could be defined. This is the first time that the function of Atg9 is uncoupled from that of the other core Atgs in any autophagy process. In macro-ER-phagy, Atg9 is required for ERAM formation and is present on these membranes. Interestingly, in the absence of over-expressed cargo, a large portion of peripheral Atg9 structures co-localize with ER [43].

Ypt1 regulates ER-to-Golgi transport and all types of macro-autophagy in the context of different GEF-GTPase-effector modules [23]. Even though we established the *ypt1-1* mutation affects autophagy and not ER-to-Golgi transport [21,22], it was important to ensure that mutations in other ER-to-Golgi and Golgi-to-vacuole regulators do not exhibit macro-ER-phagy phenotypes. Sec12 mediates exit from the ER towards the Golgi. In *sec12-ts* mutant cells, some membrane proteins are trapped in the ER, and ER stress is high. However, these mutant cells are not defective in macro-ER-phagy because excess membrane proteins can reach the vacuole, and *atg9∆* enhances their phenotype rather than masking it. In addition, inhibition of transport from the Golgi or endosomes in *vps4∆* mutant cells did not affect macro-ER-phagy. Moreover, even though the macro-ER-phagy phenotype of *ypt1-1* is more severe than deletion of any single Atg, the observation that the phenotype of the *atg11∆ atg17* double mutant is similar to that of *ypt1-1* suggests that the *ypt1-1* phenotype is caused by a defect in autophagy. All these results support the idea that the function of Ypt1 in macro-ER-phagy is unrelated to its function in ER-to-Golgi transport.

Vps21 was used here to show that macro-ER-phagy cargo utilizes APs like other macro-autophagy pathways. We have recently shown that Vps21 is required for specific and nonspecific autophagy, and *vps21∆* mutant cells accumulate autophagic structures marked by Atg8 [24]. Here we show that Vps21 also plays a role in macro-ER-phagy because a macro-ER-phagy cargo accumulates in Atg8-marked autophagic structures of *vps21∆* mutant cells. The observation that this phenotype can be masked by *ypt1-1* supports the idea that Ypt1 functions before Vps21 in this pathway.

While UPR is induced when the second step of macro-ER-phagy is blocked, it is not required for this process. This idea is based on the observation that deletion of *IRE1* or *HAC1*, which completely abolish the UPR, does not affect macro-ER-phagy in *YPT1* (WT) cells, and can uncouple UPR from the formation of ERAM in *ypt1-1* mutant cells. While it is not clear why UPR is induced in mutant cells defective in the second step of macro-ER-phagy (e.g., *ypt1-1*, *atg1∆*, *atg8∆* and *atg11∆*), the finding that Kar2 (BiP) is excluded from ERAM in *ypt1-1* mutant cells supports the model that sequestration of Kar2 from Ire1 (which presumably is retained) is one of the requirements for UPR induction [44].

### Open questions

While overexpression of integral PM proteins was used here to detect clear defects in the pathway, partial defects in delivery of some ER-resident proteins to the vacuole were observed without it in *ypt1-1* mutant cells [17]. Moreover, we show here that some resident ER proteins are delivered to the vacuole through basal macro-ER-phagy also in wild type cells, and overexpression of a single integral PM protein further induces this pathway. This suggests that macro-ER-phagy is a constitutive ERQC process that clears excess membrane proteins from the ER. Our results also imply the existence of a complementary process that ensures that the steady state level of certain resident ER proteins is maintained when macro-ER-phagy is induced by over-expression of membrane proteins. While it is not clear whether this steady state level is regulated at the level of mRNA or protein, it seems that it does not involve UPR, which regulates the expression of multiple genes [45], because UPR is not induced in wild type cells overexpressing integral-membrane proteins (Figs 5C and S3C). Specific mechanisms that underlie basal and induced macro-ER-phagy and the regulation of ER-resident proteins level when macro-ER-phagy is induced, need to be deciphered.

Other open questions to be addressed in the future are how macro-ER-phagy cargo is recognized and sorted to ERAM and the relationship between this pathway and ERAD. We propose that macro-ER-phagy cargo is recognized either on the cytoplasmic side of the ER or in the ER membrane, because at least one cargo, GFP-Snc1-PEM, does not have a lumenal domain. Under ER stress, the ERAD-M and ERAD-C pathways recognize misfolded intra-membrane and cytosolic domains of membrane proteins, respectively [46]. While it is possible that excess integral-membrane proteins, especially when tagged with a fluorescence moiety, are partially misfolded, it seems that ERAD does not play a major role in their clearance because deletion of Ire1 and Hac1, which are required for ERAD induction, does not affect this clearance. To determine whether ERAD plays a role in the degradation of excess ER membrane proteins, the effect of ERAD mutations, alone and in combination with autophagy mutations, on this process should be determined in future experiments. It is intriguing if and how proteins are sorted between macro-ER-phagy and ERAD for degradation, whether they are sequestered to different domains, and whether yet unknown ER-phagy-specific Atgs play a role in this process. In addition, cooperation between ER-phagy and other ERQC processes has been proposed [4]. The idea is that each pathway can serve as a backup for the other. The synergistic effect of overexpression of multiple membrane proteins in *atg11∆* mutant cells supports this idea. We propose that these mutant cells cope with excess of a single protein using another ERQC pathway, possibly ERAD, while overexpression of two proteins saturates this outlet.

Another question is whether ER-exit sites towards the Golgi (ERES) and ER-phagy overlap. Recently, ERES were proposed to be the site of AP biogenesis [47]. Our result that Sec12, an ERES integral-membrane protein, is not delivered to the vacuole through macro-ER-phagy, does not support a role for ERES in this process.

What is the relevance of degradation of excess membrane proteins by macro-ER-phagy? First, specific impairment of macro-ER-phagy should be considered in studies that require overexpression of membrane proteins. For example, ERQC was identified as the bottleneck of overexpression of heterologous proteins in yeast. Co-overexpression of chaperons, like BiP, did not affect this block [48], supporting the idea that macro-ER-phagy, and not ERAD, is the likely cause of this bottleneck. Most importantly, overexpression of membrane proteins has been associated with multiple human diseases. For example, amplification of HER2, a human epidermal growth factor receptor, occurs in ~20% of breast cancers and is associated with a more aggressive disease [49,50]. Likewise, overexpression of the M oncostatin receptor is associated with increased aggressiveness of cervical cancer and is considered a therapeutic target [51]. Moreover, overexpression of the P-glycogen efflux pump is associated with bone inflammation and is considered a potential therapeutic target [52]. Finally, chronic ER stress is associated with neurodegenerative diseases and therapeutic benefits of chemical autophagy activators were reported [53]. Therefore, if the mechanism we unraveled here is conserved from yeast to human like all other basic cellular processes, macro-ER-phagy of excess membrane protein would be relevant to human disease.

### Note added in proof

While this paper was under revision, Mochida et al [54] reported the identification of two new receptors, Atg39 and Atg40, required for autophagy of the ER during nitrogen starvation, which induces non-selective autophagy. Interestingly, Atg40 is similar to the FM134B reticulon, an autophagy receptor of ER in mammals, which was implicated in neuropathy in humans [55]. Future studies should clarify whether these new receptors play a role in macro-ER-phagy of ER proteins under normal growth conditions described here.

## Materials and Methods

### Strains, plasmids, construction and reagents

Details about strains, plasmids and reagents used in this manuscript are given in Supplementary Experimental Procedures (S1 File), and S1 and S2 Tables. Construction of strains and plasmids is described in Supplementary Experimental Procedures (S1 File).

### Yeast culture conditions

All yeast strains were grown in rich (YPD) or minimal (SD) media containing the necessary amino acid supplements. In tunicamycin experiments, cells were incubated with 5 μg/ml tunicamycin for 1.5 hours before collecting them. Experiments involving DTT treatment were performed as previously described [15].

### Protein level analysis

The protein level of GFP-Snc1-PEM, HA-tagged Sec61, Sec13 and Sec12 was determined as previously described [17]. The protein level of mCherry-tagged Hmg1 and Kar2 was determined according to the previously described methods [56,57]. Quantification was done using ImageJ and adjusted to the loading control.

### Microscopy

For live-cell microscopy, cells expressing fluorescently tagged proteins were grown to mid-log phase in appropriate media. Fluorescence microscopy was performed using deconvolution Axioscope microscope (Carl Zeiss, Thornwood, NY) with FITC (GFP, yEGFP) and TexasRed (mCherry) sets of filters. Labeling of vacuole membranes with FM4-64 was done using a 5 min pulse followed by 60 min chase as previously described [58]. Labeling the vacuolar lumen with CMAC-Arg was done according to the manufacturer’s instructions. Immunofluorescence microscopy of yeast cells was performed as previously described [59].

### UPR β-gal assay

The β-gal assay was done with cells as previously described [17]. Briefly, cells were transformed with a plasmid (pJC104) expressing the LacZ gene behind four UPR elements [60]. The level of β-galactosidase in cell extracts was determined as β-gal (Miller) units [61]. The values are 0.6–1.0 units for WT cells. Graphs represent percent of UPR in experimental cells (e.g., mutant cells) as fold of β-Gal units in WT cells; averages and error bars (represent STDEV) were calculated from two independent reactions; each result represents at least two different experiments from independent transformants. Statistical significance (p value) was calculated from two different experiments using independent transformants, each with two independent reactions (total of four reactions). In our experiments, tunicamycin treatment (5 μg/ml for 90 min) results in a 12-22-fold induction over untreated WT cells.

## Supporting Information

S1 Fig Deletion of Atg17, but not of selective Atgs, exacerbates the macro-ER-phagy phenotypes of *atg11∆* mutant cells. **A-C.** Whereas deletion of *ATG17* in wild-type cell does not result in a macro-ER-phagy defect, it exacerbates the *atg11∆* mutant phenotype. The shown phenotypes: increase of GFP-Snc1-PEM protein level (**A**), accumulation of aberrant intracellular GFP-Snc1-PEM structures (**B**), and induction of the UPR response (**C**). Wild type (WT), *atg17∆*, *atg11∆*, *atg17∆ atg11∆*, and *ypt1-1* (for comparison) mutant cells overexpressing GFP-Snc1-PEM were analyzed as described In Fig 1 legend. **D-F.** Deletion of Atg11 together the other known selective Atgs required for the CVT pathway (Atg19), mitophagy (Atg32) and pexophagy (Atg36), results in phenotypes similar to those of *atg11∆* mutant cells: increase of GFP-Snc1-PEM protein level (**D**), accumulation of aberrant intracellular GFP-Snc1-PEM structures (**E**), and induction of the UPR response (**F**). Wild type (WT), *atg19∆*, *atg32∆*, and *atg36∆* mutant cells, without (-) and with (+) *atg11∆*, overexpressing GFP-Snc1-PEM were analyzed as described In Fig 1 legend. B and E: shown from top to bottom: DIC, GFP and % cells with intracellular GFP-Snc1-PEM. +/- and error bars represent STDEV. Results in this figure represent at least two independent experiments.
(PDF)

S2 Fig The macro-ER-phagy defect of *atg9∆* mutant cells is different from that of *atg11∆* and Atg9 is epistatic to Atg1. **A.** GFP-Snc1-PEM structures that accumulate in *atg9∆* mutant cells co-localize with the ER marker Sec61. Endogenous Sec61 was tagged with mCherry in WT and *atg9∆* mutant cells. Accumulation of overexpressed GFP-Snc1-PEM and its co-localization with Sec61 were determined using live-cell microscopy. Shown from left to right: DIC, GFP, mCherry, merge, % cells with GFP-Snc1 in aberrant intracellular structures, % cells in which the GFP-Snc1 structures co-localize with Sec61-mCherry, and the ratio of intracellular GFP-Snc1-PEM / PM. Arrows point to co-localization. **B.** Overexpression of GFP-Snc1-PEM in *atg9∆*, but not in WT and *atg11∆*, mutant cells results in a growth defect. The growth rate of WT (left), *atg11∆* (middle) and *atg9∆* (right) mutant cells, transformed with empty plasmid (empty symbol) or a plasmid overexpressing GFP-Snc1-PEM (filled symbol), in selective minimal (SD) medium was determined by measuring OD_600_ over time. **C-E.** Atg9 is epistatic to Atg1. Wild type (WT), *atg9∆*, *atg1∆*, and *atg9∆ atg1∆* mutant cells overexpressing GFP-Snc1-PEM were analyzed as described for Fig 1A–1C, respectively. The shown phenotypes: increase of GFP-Snc1-PEM protein level (**C**), accumulation of aberrant intra-cellular GFP-Snc1-PEM structures (**D**), and induction of UPR (**E**). Whereas Snc1-PEM accumulates in *atg9∆* (single) and *atg9∆ atg1∆* (double) mutant cells to a level similar to that of *atg1∆*, only ~20% of these mutant cells accumulate it in intracellular aberrant structures, and UPR is not induced in *atg9∆* single or double mutant cells. +/- and error bars represent STDEV. Results in this represent at least two independent experiments.
(PDF)

S3 Fig Cells defective in the ER-exit regulator Sec12 accumulate GFP-Snc1-PEM in their ER and induce UPR even without its overexpression. The effects of overexpression of GFP-Snc1-PEM in WT, *sec12ts* and *ypt1-1* (for comparison) were determined as described for Fig 1A–1C, respectively. The shown phenotypes: increase of GFP-Snc1-PEM protein level (**A**), accumulation of GFP-Snc1-PEM in the ER (**B**), and induction of UPR (**C**). **A.** The level of GFP-Snc1-PEM increases ~3.5 fold in *sec12ts* mutant cells when compared to WT. **B.** ~50% of the *sec12ts* mutant cells accumulate GFP-Snc1 PEM in their ER and almost all cells contain aberrant Sec61-labeled structures. Endogenous Sec61 was tagged with mCherry in WT, *sec12ts* and *ypt1-1* mutant cells. Shown from left to right: DIC, GFP, mCherry, merge, % cells with aberrant Snc1-PEM structures, % cells with aberrant Sec61 structures, and % cells in which the aberrant Snc1-PEM localizes in the ER (co-localization). **C.** UPR is induced in *sec12ts* mutant cells even without overexpression of GFP-Snc1-PEM. The effect of overexpression of Snc1-PEM on the UPR was determined in WT, *sec12ts* and *ypt1-1* (for comparison). Overexpression of Snc1-PEM results in increased UPR in *ypt1-1* (p = 0.005), but decreased UPR in *sec12ts*, mutant cells (p = 0.05). UPR can be further induced in both *sec12ts* and *ypt1-1* mutant cells overexpressing GFP-Snc1-PEM by tunicamycin (as described in Fig 2E). +/- and error bars represent STDEV. Results in this figure represent at least two independent experiments.
(PDF)

S4 Fig Vps4 is not required for macro-ER-phagy and Ypt1 is not required for micro-ER-phagy. **A-C.** *vps4∆* mutant cells are not defective in macro-ER-phagy. GFP-Snc1-PEM was overexpressed in WT and *vps4∆* mutant cells and the following phenotypes were tested as described for Fig 1A–1C, respectively: increase in the level of GFP-Snc1-PEM protein (**A,** *atg9∆*, *atg11∆* and *atg1∆* are shown as positive controls), accumulation of GFP-Snc1-PEM in aberrant structures (**B**), and induction of the UPR response (**C,** *atg11∆* is shown as a positive control). **B.** The ER marker Sec61 was tagged at its C-terminus with mCherry in WT and *vps4∆* mutant cells. Shown from top to bottom: DIC, GFP, mCherry, merge, % cells with intracellular Snc1-PEM (number of cells with internal GFP / number of cells visualized), and % cells in which intra-cellular Snc1-PEM co-localizes with Sec61. In all three assays, *vps4∆* mutant cells behave like WT. **D.** *ypt1-1* mutant cells are not defective in micro-ER-phagy. The vacuolar peptidases Pep4 and Prb1 were deleted in WT (*YPT1*) and *ypt1-1* mutant cells in which Sec61 was tagged at its C-terminus with yEGFP. The cells were stained with FM4-64 to label the vacuolar membrane. DTT (8 mM) was added for 4 hours to induce “ER whorls” in the vacuole [15]. No whorls were seen in WT and *ypt1-1* mutant cells not treated with DTT (top). Whorls were detected in 50% of WT and 59% of *ypt1-1* mutant cells treated with DTT (bottom). Shown from left to right: DIC, GFP, FM4-64, merge, and % cells with Sec61-GFP whorls in the FM4-64 labeled vacuole. +/- and error bars represent STDEV. Results in this figure represent at least two independent experiments.
(PDF)

S5 Fig Effect of co-overexpression of two macro-ER-phagy cargos. **A.** Quantification of live-cell microscopy results shown in Fig 5E. Shown for each strain, from left to right: % cells with intracellular Snq2, % cells with intracellular Snc1-PEM and % cells in which they co-localize (relevant only for the co-overexpression). **B.** Effect of overexpression of ER-phagy cargos on WT (left) and *atg11∆* mutant cells (right). Cells were transformed with two empty plasmids (black), one empty and one overexpressing Snq2-yEGFP (green), one empty and one overexpressing DsRed-Snc1-PEM (red), or two plasmids overexpressing both cargos (yellow). The growth of cells in selective minimal (SD) medium was determined by measuring OD_600_ over time. The growth of WT cells was not affected by overexpression of either or both cargos. However, whereas the growth of *atg11∆* mutant cells expressing one cargo was also not affected, overexpression of both cargos resulted in a slower growth. +/- and error bars represent STDEV. Results in this figure represent at least two independent experiments.
(PDF)

S6 Fig Immuno-blot analysis of ER-resident proteins with and without overexpression of GFP-Snc1-PEM. The levels of ER-resident proteins were determined by immuno-blot analysis in cells that either do not (**A-C**) or do overexpress GFP-Snc1-PEM (**E-G**). The analysis was done as described in Fig 6 legend. **A-C:** Hmg1 (**A**), Sec12-3xHA (**B**) and Kar2 (**C**) expressing cells (2 independent un-transformed colonies) were tested by immuno-blot analysis (using anti-Hmg1, anti-HA and anti-Kar2 antibodies, respectively). Shown from top to bottom: strain genotype, the specific ER-resident protein tested protein, G6PDH (loading control), quantification of ER-resident protein expressed as average fold of WT. **D.** Stability of Sec61 protein after addition of cycloheximide in cells with and without GFP-Snc1-PEM overexpression. Cycloheximide (75 μ g/ml) was added to cells from Fig 6E and cell extracts were made at the times shown. The level of Sec61 was determined by immuno-blot analysis and presented in a graph showing Sec61 level as % of time zero at different times (min) after addition of cycloheximide. Sec61 is degraded seven-times faster in cells over-expressing GFP-Snc1-PEM. **E.** The level of the ER-resident protein Hmg1 is similar whether GFP-Snc1-PEM is overexpressed or not. The experiment was done as described for Fig 6E. Shown from top to bottom: plasmid, Hmg1, G6PDH (loading control), GFP-Snc1-PEM, and quantification of Hmg1 expressed as average fold of WT with empty plasmid. **F-H**: Strains were transformed with a 2μ plasmid for overexpression of GFP-Snc1-PEM. Immuno-blot analysis was performed for GFP-Snc1-PEM (using anti-GFP antibodies) and for the different ER-resident proteins: **F.** Hmg1-mCherry (using anti-Hmg1 antibodies), **G.** Sec12-3xHA (using anti-HA antibodies), and **H.** Kar2 (using anti-Kar2 antibodies). In each panel, shown from top to bottom: strain genotype, the specific ER-resident protein tested, quantification of the ER-resident protein bands compared to WT, GFP-Snc1-PEM, quantification of the GFP-Snc1-PEM bands compared to WT, and G6PDH (loading control). Results from four strains for each ER-resident protein were used for Fig 6D (no overexpression) and 6H (GFP-Snc1-PEM overexpression). +/- represent STDEV. Results in this figure represent at least two independent experiments.
(PDF)

S7 Fig Live-cell microscopy analysis of ER-resident proteins upon overexpression of GFP-Snc1-PEM. **A.** Whereas Sec61 co-localizes with 60% of the aberrant GFP-Snc1-PEM intracellular structures in cells defective in vacuolar proteolysis, Sec12 and Kar2 do not. The experiment was done as described for Fig 7A, except that Kar2 was analyzed by immunofluorescence microscopy using anti-Kar2 antibodies (36–44 cells were analyzed for each strain). Results from this panel were used in Fig 7D (left). **B.** >80% of *ypt1-1* mutant cells, *PEP4 PRB1* and *ypt1-1 pep4∆ prb1∆*, accumulate intracellular Snc1-PEM structures. Sec61 co-localizes with ~85% of the Snc1-PEM structures in these mutant cells, Sec12 with ~38% and Kar2 with ~11% (36–44 cells were analyzed for each strain). The experiment was done as described for Fig 7C except that Kar2 was analyzed by immunofluorescence microscopy using anti-Kar2 antibodies. Results from this panel were used in Fig 7D (right). +/- represent STDEV. Results in this figure represent at least two independent experiments.
(PDF)

S1 File Supplementary Experimental Procedures. Strains, plasmids and reagents. Plasmid and strain construction.
(DOC)

S1 Table Yeast strains used in this study. (DOC)

S2 Table Plasmids used in this study. (DOC)
