# Supplementary figures and images for: A Role for Macro-ER-Phagy in ER Quality Control
Source: PLoS Genet. 2015 Jul 16;11(7):e1005390. doi: 10.1371/journal.pgen.1005390 (PMC4504476; doi:10.1371/journal.pgen.1005390)

**Figure S1**

**A.**

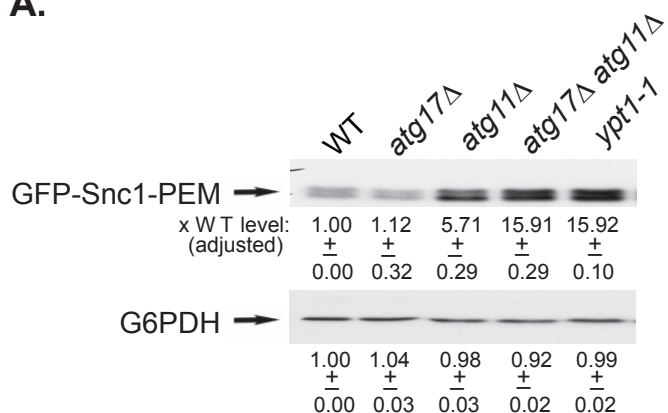

**B.**

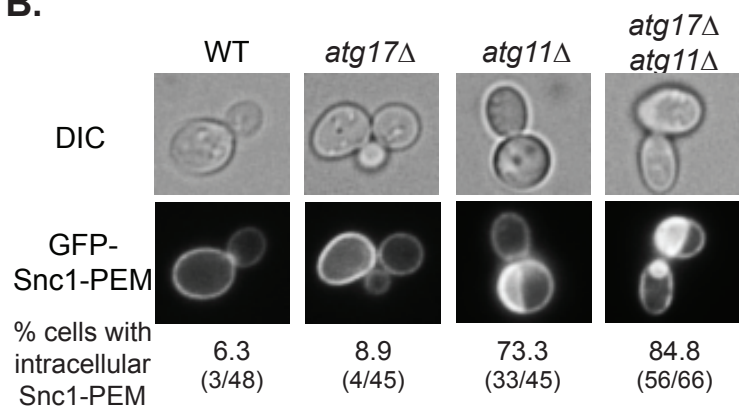

**C.**

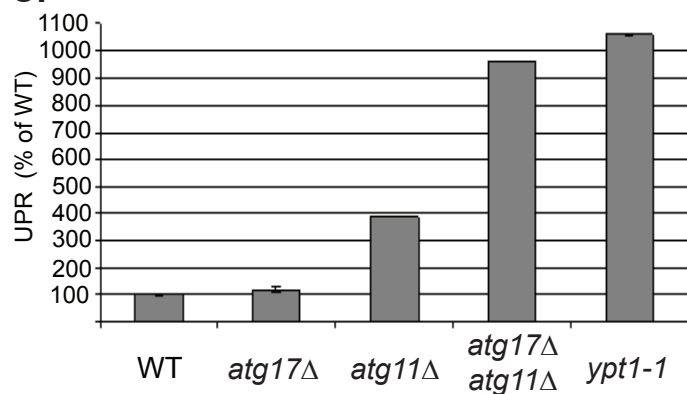

**D.**

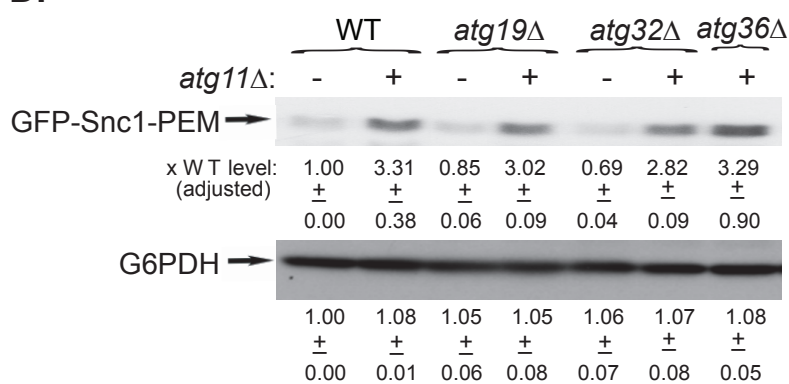

**E.**

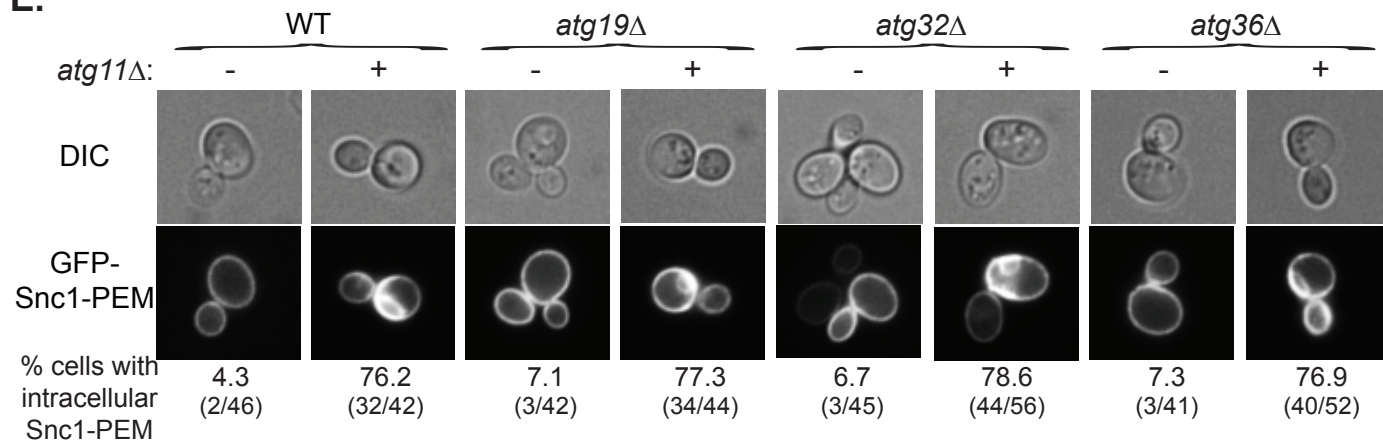

**F.**

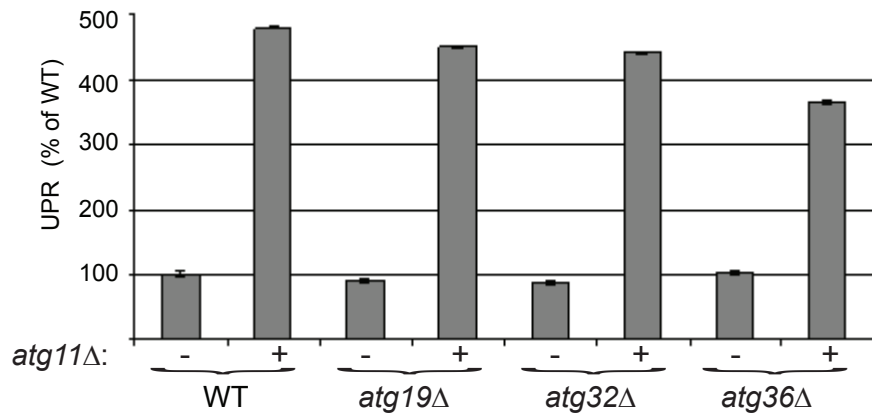

Supplement: S1 Fig — A-C. Whereas deletion of ATG17 in wild-type cell does not result in a macro-ER-phagy defect, it exacerbates the atg11∆ mutant phenotype. The shown phenotypes: increase of GFP-Snc1-PEM protein level (A), accumulation of aberrant intracellular GFP-Snc1-PEM structures (B), and induction of the UPR response (C). Wild type (WT), atg17∆, atg11∆, atg17∆ atg11∆, and ypt1-1 (for comparison) mutant cells overexpressing GFP-Snc1-PEM were analyzed as described In Fig 1 legend. D-F. Deletion of Atg11 together the other known selective Atgs required for the CVT pathway (Atg19), mitophagy (Atg32) and pexophagy (Atg36), results in phenotypes similar to those of atg11∆ mutant cells: increase of GFP-Snc1-PEM protein level (D), accumulation of aberrant intracellular GFP-Snc1-PEM structures (E), and induction of the UPR response (F). Wild type (WT), atg19∆, atg32∆, and atg36∆ mutant cells, without (-) and with (+) atg11∆, overexpressing GFP-Snc1-PEM were analyzed as described In Fig 1 legend. B and E: shown from top to bottom: DIC, GFP and % cells with intracellular GFP-Snc1-PEM. +/- and error bars represent STDEV. Results in this figure represent at least two independent experiments. (PDF) [file pgen.1005390.s001.pdf]

Figure S2

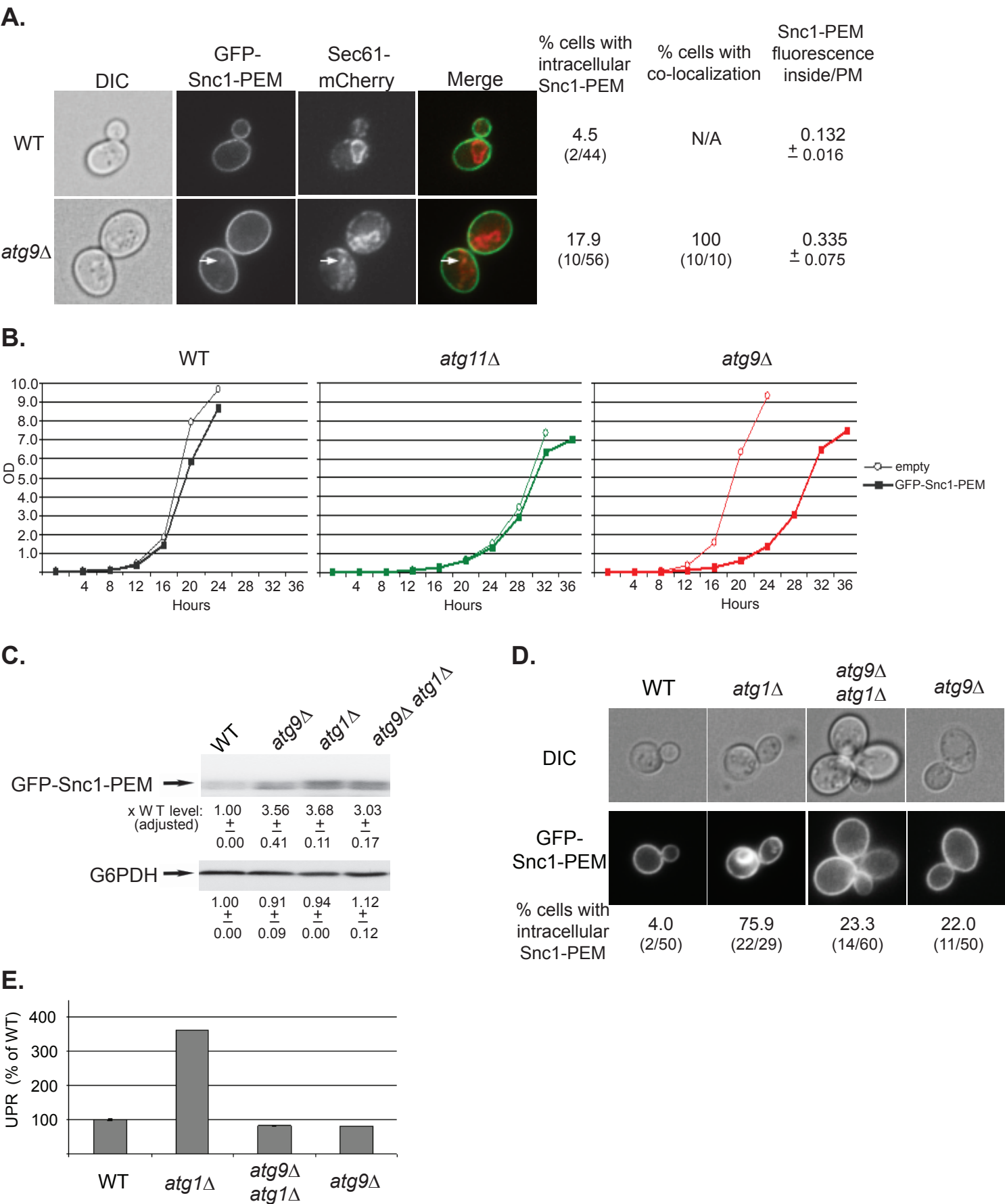

Supplement: S2 Fig — A. GFP-Snc1-PEM structures that accumulate in atg9∆ mutant cells co-localize with the ER marker Sec61. Endogenous Sec61 was tagged with mCherry in WT and atg9∆ mutant cells. Accumulation of overexpressed GFP-Snc1-PEM and its co-localization with Sec61 were determined using live-cell microscopy. Shown from left to right: DIC, GFP, mCherry, merge, % cells with GFP-Snc1 in aberrant intracellular structures, % cells in which the GFP-Snc1 structures co-localize with Sec61-mCherry, and the ratio of intracellular GFP-Snc1-PEM / PM. Arrows point to co-localization. B. Overexpression of GFP-Snc1-PEM in atg9∆, but not in WT and atg11∆, mutant cells results in a growth defect. The growth rate of WT (left), atg11∆ (middle) and atg9∆ (right) mutant cells, transformed with empty plasmid (empty symbol) or a plasmid overexpressing GFP-Snc1-PEM (filled symbol), in selective minimal (SD) medium was determined by measuring OD600 over time. C-E. Atg9 is epistatic to Atg1. Wild type (WT), atg9∆, atg1∆, and atg9∆ atg1∆ mutant cells overexpressing GFP-Snc1-PEM were analyzed as described for Fig 1A–1C, respectively. The shown phenotypes: increase of GFP-Snc1-PEM protein level (C), accumulation of aberrant intra-cellular GFP-Snc1-PEM structures (D), and induction of UPR (E). Whereas Snc1-PEM accumulates in atg9∆ (single) and atg9∆ atg1∆ (double) mutant cells to a level similar to that of atg1∆, only ~20% of these mutant cells accumulate it in intracellular aberrant structures, and UPR is not induced in atg9∆ single or double mutant cells. +/- and error bars represent STDEV. Results in this represent at least two independent experiments. (PDF) [file pgen.1005390.s002.pdf]

**Figure S3**

**A.**

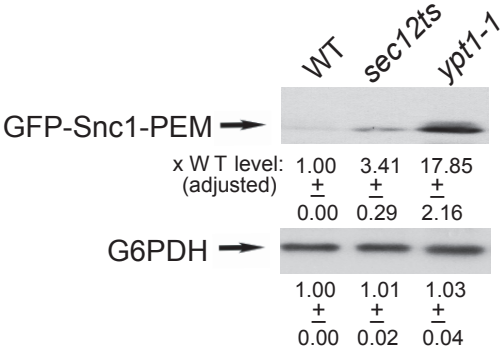

**B.**

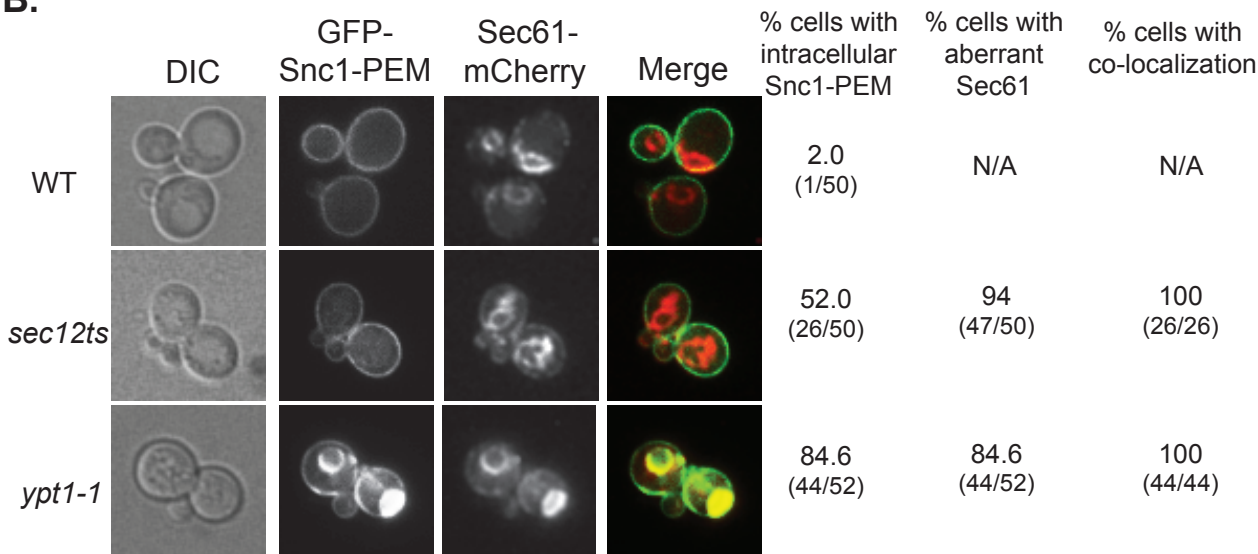

**C.**

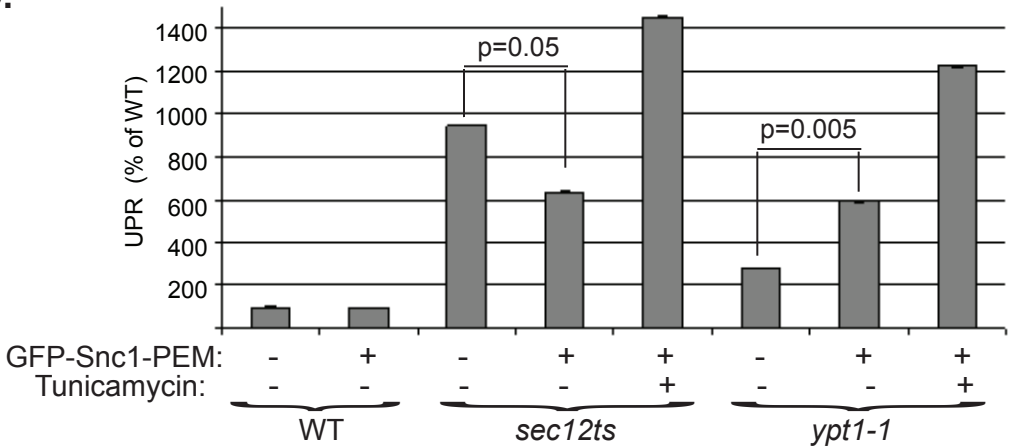

Supplement: S3 Fig — The effects of overexpression of GFP-Snc1-PEM in WT, sec12ts and ypt1-1 (for comparison) were determined as described for Fig 1A–1C, respectively. The shown phenotypes: increase of GFP-Snc1-PEM protein level (A), accumulation of GFP-Snc1-PEM in the ER (B), and induction of UPR (C). A. The level of GFP-Snc1-PEM increases ~3.5 fold in sec12ts mutant cells when compared to WT. B. ~50% of the sec12ts mutant cells accumulate GFP-Snc1 PEM in their ER and almost all cells contain aberrant Sec61-labeled structures. Endogenous Sec61 was tagged with mCherry in WT, sec12ts and ypt1-1 mutant cells. Shown from left to right: DIC, GFP, mCherry, merge, % cells with aberrant Snc1-PEM structures, % cells with aberrant Sec61 structures, and % cells in which the aberrant Snc1-PEM localizes in the ER (co-localization). C. UPR is induced in sec12ts mutant cells even without overexpression of GFP-Snc1-PEM. The effect of overexpression of Snc1-PEM on the UPR was determined in WT, sec12ts and ypt1-1 (for comparison). Overexpression of Snc1-PEM results in increased UPR in ypt1-1 (p = 0.005), but decreased UPR in sec12ts, mutant cells (p = 0.05). UPR can be further induced in both sec12ts and ypt1-1 mutant cells overexpressing GFP-Snc1-PEM by tunicamycin (as described in Fig 2E). +/- and error bars represent STDEV. Results in this figure represent at least two independent experiments. (PDF) [file pgen.1005390.s003.pdf]

**Figure S4**

**A.**

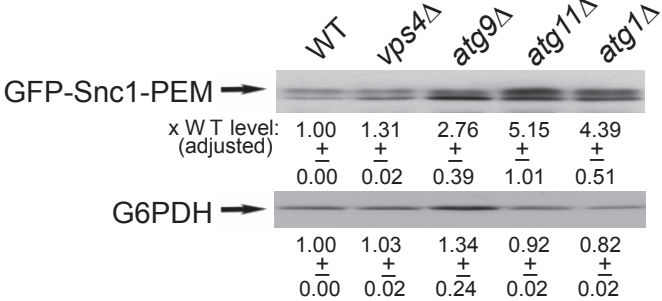

**B.**

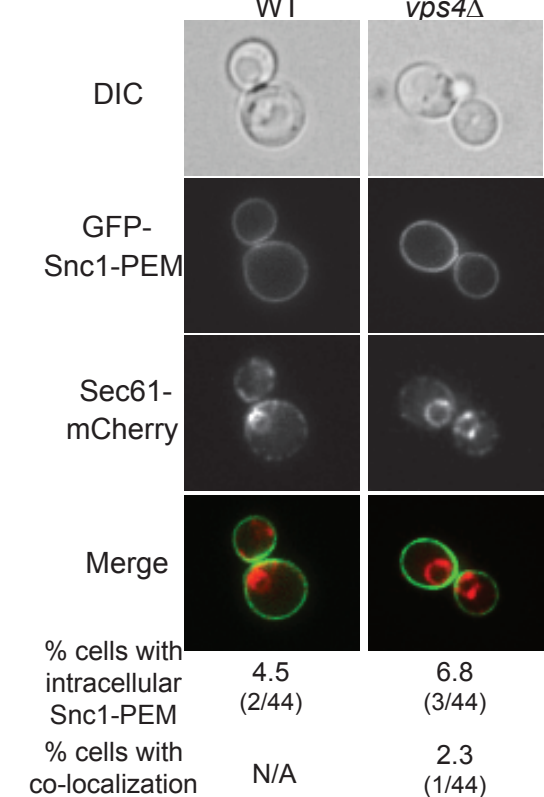

**C.**

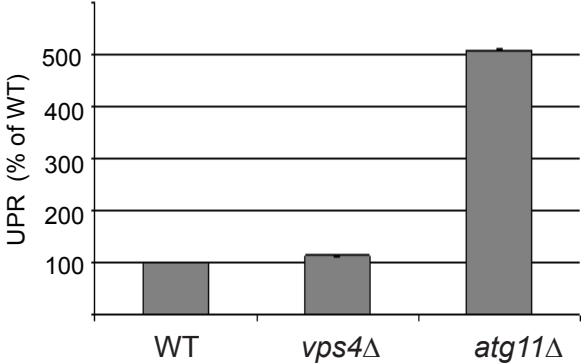

**D.**

+DTT

*YPT1 pep4Δprb1Δ*  
*ypt1-1 pep4Δprb1Δ*  
*YPT1 pep4Δprb1Δ*  
*ypt1-1 pep4Δprb1Δ*

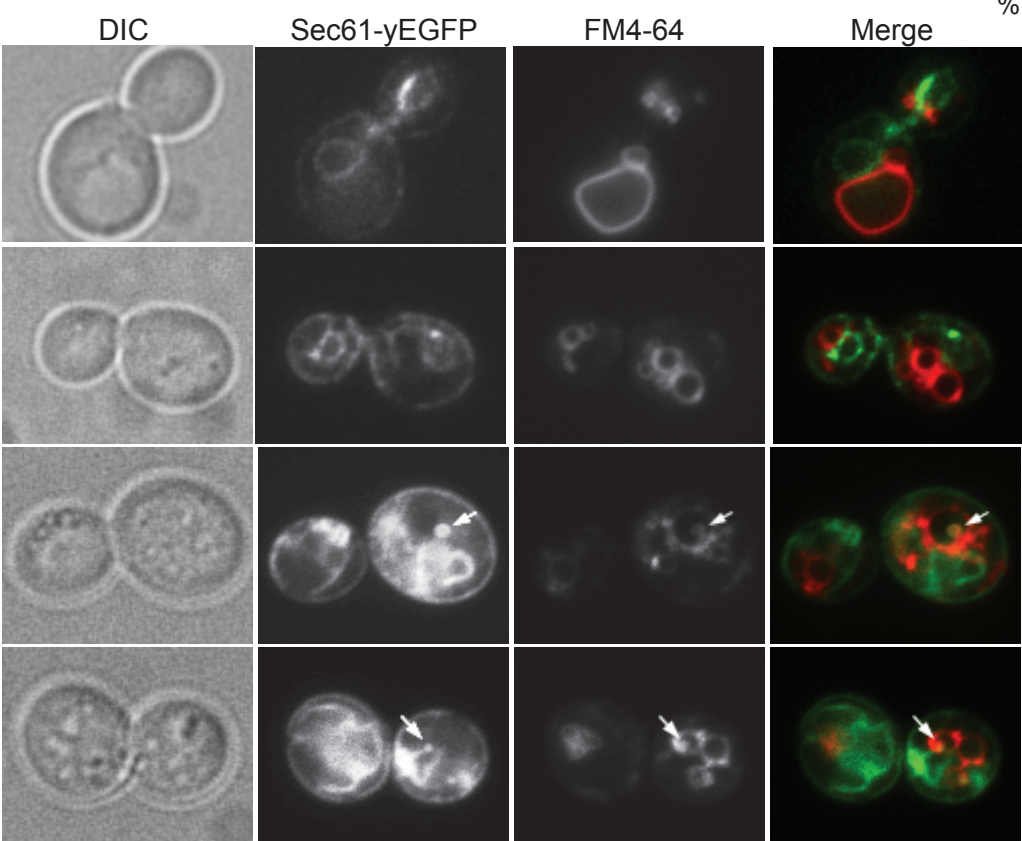

% cells with whorls  
0.00 (0/22)  
0.00 (0/33)  
50.00 (10/20)  
59.09 (13/22)

Supplement: S4 Fig — A-C. vps4∆ mutant cells are not defective in macro-ER-phagy. GFP-Snc1-PEM was overexpressed in WT and vps4∆ mutant cells and the following phenotypes were tested as described for Fig 1A–1C, respectively: increase in the level of GFP-Snc1-PEM protein (A, atg9∆, atg11∆ and atg1∆ are shown as positive controls), accumulation of GFP-Snc1-PEM in aberrant structures (B), and induction of the UPR response (C, atg11∆ is shown as a positive control). B. The ER marker Sec61 was tagged at its C-terminus with mCherry in WT and vps4∆ mutant cells. Shown from top to bottom: DIC, GFP, mCherry, merge, % cells with intracellular Snc1-PEM (number of cells with internal GFP / number of cells visualized), and % cells in which intra-cellular Snc1-PEM co-localizes with Sec61. In all three assays, vps4∆ mutant cells behave like WT. D. ypt1-1 mutant cells are not defective in micro-ER-phagy. The vacuolar peptidases Pep4 and Prb1 were deleted in WT (YPT1) and ypt1-1 mutant cells in which Sec61 was tagged at its C-terminus with yEGFP. The cells were stained with FM4-64 to label the vacuolar membrane. DTT (8 mM) was added for 4 hours to induce “ER whorls” in the vacuole [15]. No whorls were seen in WT and ypt1-1 mutant cells not treated with DTT (top). Whorls were detected in 50% of WT and 59% of ypt1-1 mutant cells treated with DTT (bottom). Shown from left to right: DIC, GFP, FM4-64, merge, and % cells with Sec61-GFP whorls in the FM4-64 labeled vacuole. +/- and error bars represent STDEV. Results in this figure represent at least two independent experiments. (PDF) [file pgen.1005390.s004.pdf]

**Figure S6**

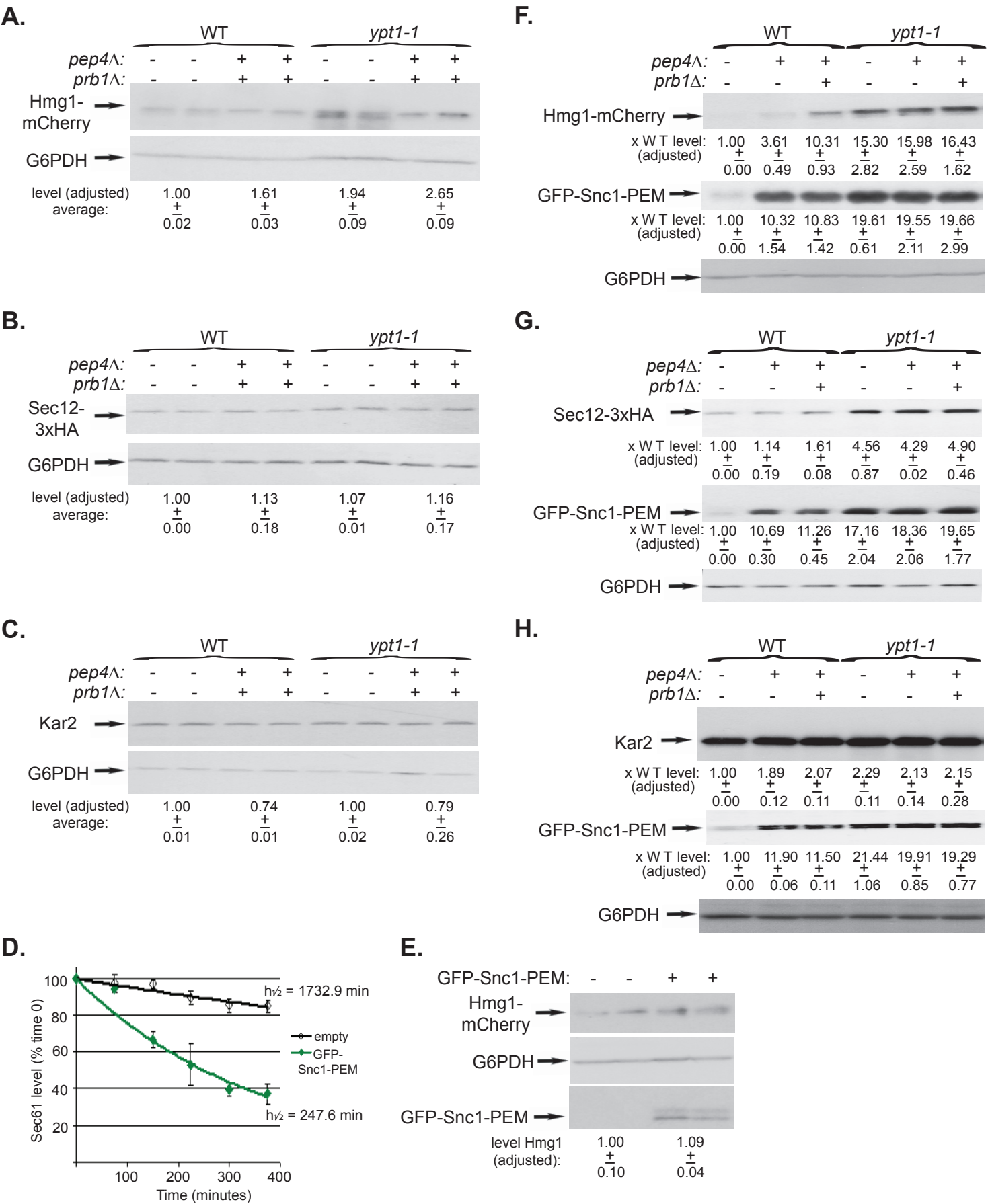

Supplement: S6 Fig — The levels of ER-resident proteins were determined by immuno-blot analysis in cells that either do not (A-C) or do overexpress GFP-Snc1-PEM (E-G). The analysis was done as described in Fig 6 legend. A-C: Hmg1 (A), Sec12-3xHA (B) and Kar2 (C) expressing cells (2 independent un-transformed colonies) were tested by immuno-blot analysis (using anti-Hmg1, anti-HA and anti-Kar2 antibodies, respectively). Shown from top to bottom: strain genotype, the specific ER-resident protein tested protein, G6PDH (loading control), quantification of ER-resident protein expressed as average fold of WT. D. Stability of Sec61 protein after addition of cycloheximide in cells with and without GFP-Snc1-PEM overexpression. Cycloheximide (75 μ g/ml) was added to cells from Fig 6E and cell extracts were made at the times shown. The level of Sec61 was determined by immuno-blot analysis and presented in a graph showing Sec61 level as % of time zero at different times (min) after addition of cycloheximide. Sec61 is degraded seven-times faster in cells over-expressing GFP-Snc1-PEM. E. The level of the ER-resident protein Hmg1 is similar whether GFP-Snc1-PEM is overexpressed or not. The experiment was done as described for Fig 6E. Shown from top to bottom: plasmid, Hmg1, G6PDH (loading control), GFP-Snc1-PEM, and quantification of Hmg1 expressed as average fold of WT with empty plasmid. F-H: Strains were transformed with a 2μ plasmid for overexpression of GFP-Snc1-PEM. Immuno-blot analysis was performed for GFP-Snc1-PEM (using anti-GFP antibodies) and for the different ER-resident proteins: F. Hmg1-mCherry (using anti-Hmg1 antibodies), G. Sec12-3xHA (using anti-HA antibodies), and H. Kar2 (using anti-Kar2 antibodies). In each panel, shown from top to bottom: strain genotype, the specific ER-resident protein tested, quantification of the ER-resident protein bands compared to WT, GFP-Snc1-PEM, quantification of the GFP-Snc1-PEM bands compared to WT, and G6PDH (loading control). Results from four stra [file pgen.1005390.s006.pdf]

Figure S7

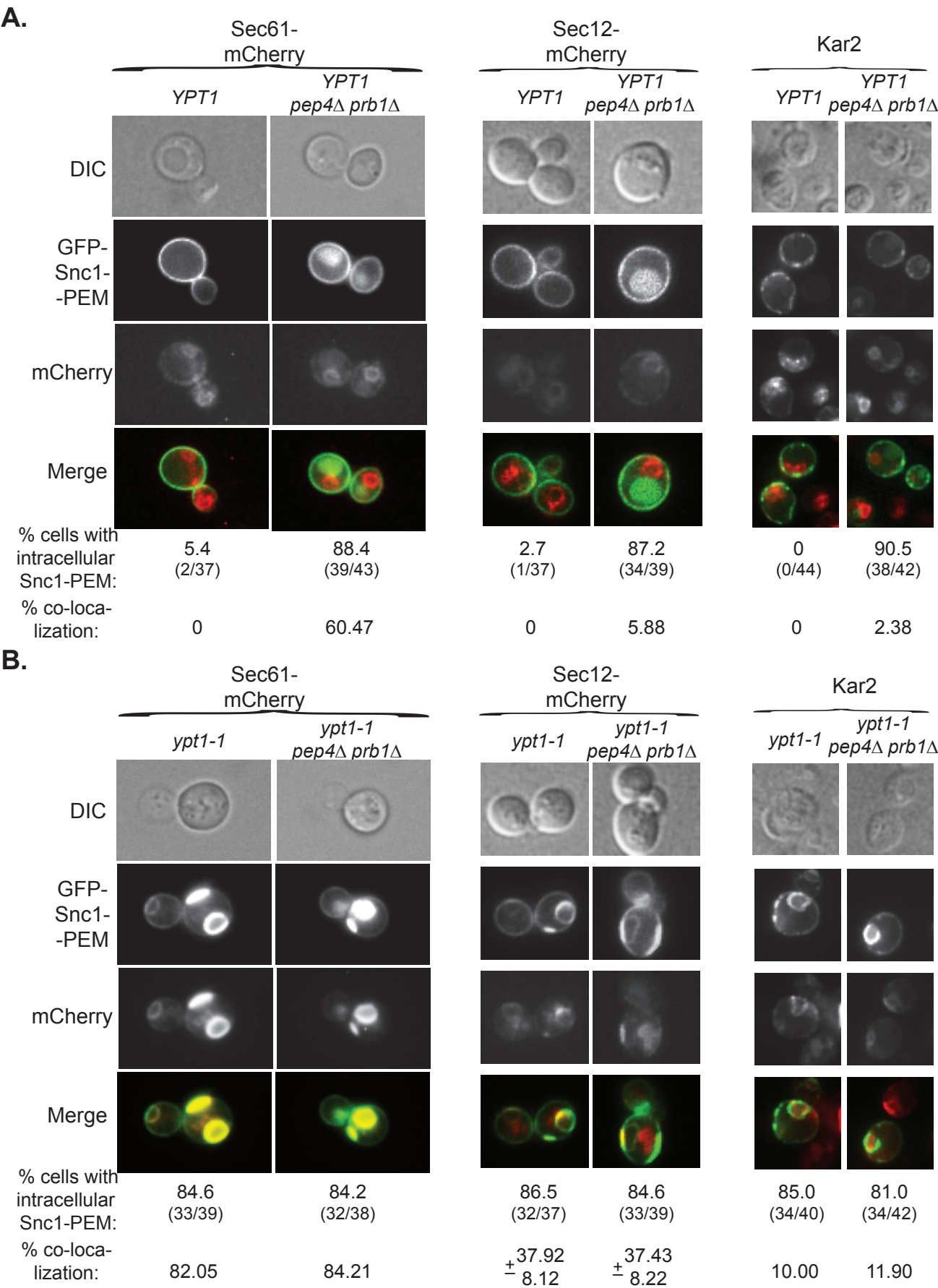

Supplement: S7 Fig — A. Whereas Sec61 co-localizes with 60% of the aberrant GFP-Snc1-PEM intracellular structures in cells defective in vacuolar proteolysis, Sec12 and Kar2 do not. The experiment was done as described for Fig 7A, except that Kar2 was analyzed by immunofluorescence microscopy using anti-Kar2 antibodies (36–44 cells were analyzed for each strain). Results from this panel were used in Fig 7D (left). B. >80% of ypt1-1 mutant cells, PEP4 PRB1 and ypt1-1 pep4∆ prb1∆, accumulate intracellular Snc1-PEM structures. Sec61 co-localizes with ~85% of the Snc1-PEM structures in these mutant cells, Sec12 with ~38% and Kar2 with ~11% (36–44 cells were analyzed for each strain). The experiment was done as described for Fig 7C except that Kar2 was analyzed by immunofluorescence microscopy using anti-Kar2 antibodies. Results from this panel were used in Fig 7D (right). +/- represent STDEV. Results in this figure represent at least two independent experiments. (PDF) [file pgen.1005390.s007.pdf]
