# Supplementary material for: A Role for Macro-ER-Phagy in ER Quality Control
Source: PLoS Genet. 2015 Jul 16;11(7):e1005390. doi: 10.1371/journal.pgen.1005390 (PMC4504476; doi:10.1371/journal.pgen.1005390)

Figure S5

A.

Strain:	WT			*atg11Δ*		
Plasmid:	% cells with intracellular Snq2	% cells with intracellular Snc1-PEM	% cells with co-localization	% cells with intracellular Snq2	% cells with intracellular Snc1-PEM	% cells with co-localization
+ Snq2- -yEGFP	6.0 (3/50)			65.5 (38/58)		
+ DsRed- -Snc1-PEM		2.2 (1/46)			52.2 (24/46)	
+ Snq2- -yEGFP + + DsRed- -Snc1-PEM	11.9 (5/42)	16.7 (7/42)	**11.9 (5/42)**	95.7 (44/46)	87.0 (40/46)	**87.0 (40/46)**

B.

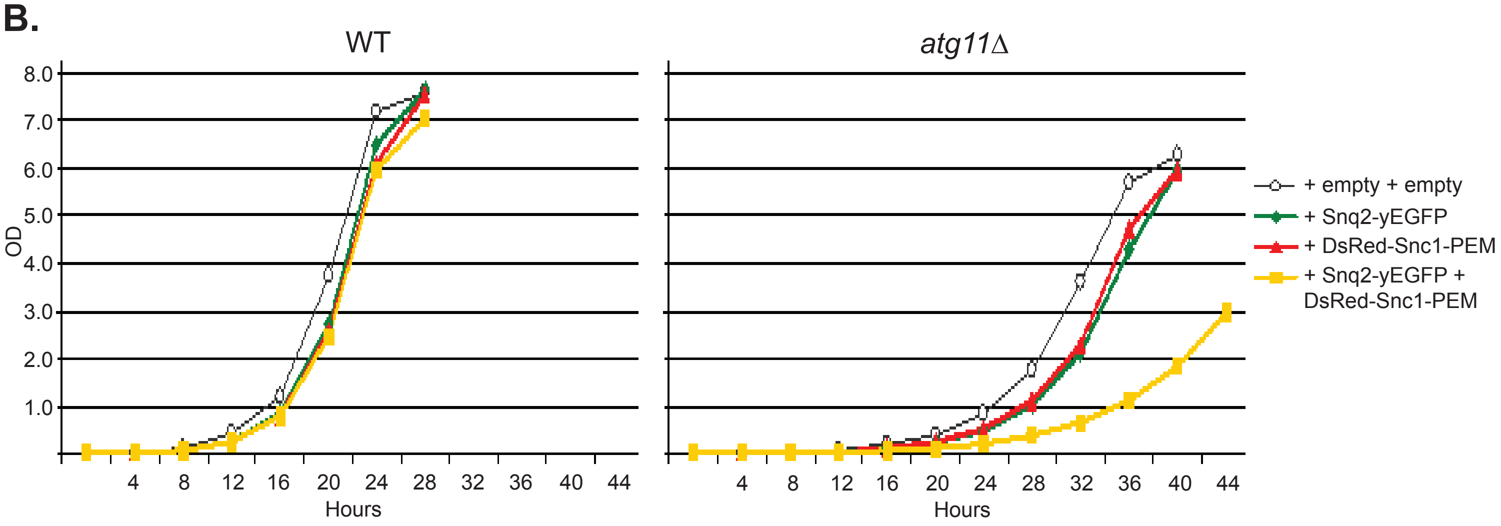

Supplement: S5 Fig — A. Quantification of live-cell microscopy results shown in Fig 5E. Shown for each strain, from left to right: % cells with intracellular Snq2, % cells with intracellular Snc1-PEM and % cells in which they co-localize (relevant only for the co-overexpression). B. Effect of overexpression of ER-phagy cargos on WT (left) and atg11∆ mutant cells (right). Cells were transformed with two empty plasmids (black), one empty and one overexpressing Snq2-yEGFP (green), one empty and one overexpressing DsRed-Snc1-PEM (red), or two plasmids overexpressing both cargos (yellow). The growth of cells in selective minimal (SD) medium was determined by measuring OD600 over time. The growth of WT cells was not affected by overexpression of either or both cargos. However, whereas the growth of atg11∆ mutant cells expressing one cargo was also not affected, overexpression of both cargos resulted in a slower growth. +/- and error bars represent STDEV. Results in this figure represent at least two independent experiments. (PDF) [file pgen.1005390.s005.pdf]
