# Supplementary material for: A Role for Macro-ER-Phagy in ER Quality Control
Source: PLoS Genet. 2015 Jul 16;11(7):e1005390. doi: 10.1371/journal.pgen.1005390 (PMC4504476; doi:10.1371/journal.pgen.1005390)
Supplement: S1 File — Strains, plasmids and reagents. Plasmid and strain construction. (DOC) [file pgen.1005390.s008.doc]

**Supplementary Experimental Procedures**

**Strains, plasmids and reagents**

Strains and plasmids used in this paper are summarized in S1 Table and S2 Table, respectively. All chemical reagents were purchased from Fisher Scientific (NJ, USA), unless otherwise noted. Media components except for amino acids were purchased from US Biological (MA, USA). Amino Acids, 2-nitrophenyl β-D-galactopyranoside, tunicamycin, and protease inhibitors were purchased from Sigma-Aldrich (St. Louis, MO). ProtoGel for western blots was purchased from National Diagnostics (GA, USA). Glass beads were purchased from BioSpec Products (Bartlesville, OK). Restriction enzymes and buffers were purchased from New England Biolabs (MA, USA). Dithiothreitol (DTT) was purchased from Invitrogen (Carlsbad, CA). FM4-64 and yeast vacuole marker sampler kit were purchased from Molecular Probes (Eugene, OR). Antibodies used in this study include mouse monoclonal anti-GFP (Roche Applied Science, Mannheim, Germany), mouse monoclonal anti-HA (Covance, WI, USA), anti-G6PDH (Sigma-Aldrich, MO, USA), goat anti-rabbit-HRP and goat anti-mouse-HRP (GE Healthcare, UK), affinity-purified rabbit anti-Hmg1 (a gift from R. Wright), anti-Snc1 (a gift from J. Gerst) and anti-Kar2 (a gift from M. Rose).

**Plasmid and strain construction**

Plasmids: To construct the plasmid for overexpression of yEGFP-tagged Snq2, first VF2 in p415-VF2 (Paquin et al, 2007) was replaced by yEGFP using BspEI and XhoI sites creating pNS1492. Then *SNQ2* ORF lacking the stop codon was cloned into the resulting plasmid using XbaI and BamHI sites generating pNS1496. Finally, the fragment containing *ADH1* promoter, *SNQ2*-yEGFP and *CYC1* terminator was subcloned into pRS425 using BssHII sites resulting in pNS1507. To construct the plasmid for overexpression of yDsRed-tagged Snc1-PEM, in vitro synthesized yeast codon optimized monomeric DsRed (Clontech Laboratories, Inc., Mountain View, CA) was cloned in p416-VF1 replacing VF1 using XbaI and BspEI sites and generating pNS1509. Then *SNC1*-PEM was cloned into BspEI and BamHI sites resulting in pNS1511. Finally, DNA fragment containing *ADH1* promoter, yDsRed-*SNC1*-PEM and *CYC1* terminator was subcloned into pRS426 using SacI and KpnI sites and creating pNS1513. To generate pNS1506, first yEGFP in pKT127 was replaced by mCherry using PacI and BssHII sites and generating pNS1505. Then KanMX4 was replaced by NatMX4 using BglII and SacI sites resulting in pNS1506.

Yeast strains: Hmg1, Sec61, Sec12 and Sec13 were tagged on the COOH-termini according to the standard technique . Deletions were done as described elsewhere . Transformations into yeast were performed by the lithium acetate method .

**References**

Baudin, A., Ozier-Kalogeropoulos, O., Denouel, A., Lacroute, F., and Cullin, C. (1993). A simple and efficient method for direct gene deletion in Saccharomyces cerevisiae. Nucleic Acids Res *21*, 3329-3330.

Ito, H., Fukuda, Y., Murata, K., and Kimura, A. (1983). Transformation of intact yeast cells treated with alkali cations. J Bacteriol *153*, 163-168.

Longtine, M.S., McKenzie, A., 3rd, Demarini, D.J., Shah, N.G., Wach, A., Brachat, A., Philippsen, P., and Pringle, J.R. (1998). Additional modules for versatile and economical PCR-based gene deletion and modification in Saccharomyces cerevisiae. Yeast *14*, 953-961.

Paquin, N., Menade, M., Poirier, G., Donato, D., Drouet, E., and Chartrand, P. (2007). Local activation of yeast ASH1 mRNA translation through phosphorylation of Khd1p by the casein kinase Yck1p. Mol Cell *26*, 795-809.

Wach, A., Brachat, A., Alberti-Segui, C., Rebischung, C., and Philippsen, P. (1997). Heterologous HIS3 marker and GFP reporter modules for PCR-targeting in Saccharomyces cerevisiae. Yeast *13*, 1065-1075.

Wach, A., Brachat, A., Pohlmann, R., and Philippsen, P. (1994). New heterologous modules for classical or PCR-based gene disruptions in Saccharomyces cerevisiae. Yeast *10*, 1793-1808.
