# Supplementary material for: A Role for Macro-ER-Phagy in ER Quality Control
Source: PLoS Genet. 2015 Jul 16;11(7):e1005390. doi: 10.1371/journal.pgen.1005390 (PMC4504476; doi:10.1371/journal.pgen.1005390)
Supplement: S1 Table — (DOC) [file pgen.1005390.s009.doc]

**S1 Table. Yeast strains used in this study**

| **Strain** | **Alias** | **Genotype** | **Source** |
| --- | --- | --- | --- |
| NSY825 | BY4741 | *Mat****a*** *leu2Δ0 ura3Δ0 his3Δ1 met15Δ0* |  |
| NSY1499 | *atg11Δ* | NSY825 *atg11Δ::KAN* | (Lipatova et al., 2012) |
| NSY1641 | *atg9Δ* | NSY825 *atg9Δ::KAN* | This study |
| NSY1642 | *atg19Δ* | NSY825 *atg19Δ::HYGRO* | This study |
| NSY1643 | *atg32Δ* | NSY825 *atg32Δ::KAN* | This study |
| NSY1644 | *atg36Δ* | NSY825 *atg36Δ::KAN* | This study |
| NSY1645 | *atg8Δ* | NSY825 *atg8Δ::KAN* | This study |
| NSY1646 | *atg1Δ* | NSY825 *atg1Δ::KAN* | This study |
| NSY1647 | *vps4Δ* | NSY825 *vps4Δ::KAN* | This study |
| NSY1648 | *vps21Δ* | NSY825 *vps21Δ::KAN* | This study |
| NSY1649 | BY4741 *SEC61-mCherry* | NSY825 *SEC61-mCherry::NAT* | This study |
| NSY1650 | *atg11Δ SEC61-mCherry* | NSY1499 *SEC61-mCherry::NAT* | This study |
| NSY1651 | *atg9Δ SEC61-mCherry* | NSY1641 *SEC61-mCherry::NAT* | This study |
| NSY1652 | *atg8Δ SEC61-mCherry* | NSY1645 *SEC61-mCherry::NAT* | This study |
| NSY1653 | *atg1Δ SEC61-mCherry* | NSY1646 *SEC61-mCherry::NAT* | This study |
| NSY1654 | *vps4Δ SEC61-mCherry* | NSY1647 *SEC61-mCherry::NAT* | This study |
| NSY128 | DBY4975 | *Matα ade2 his3-Δ200 leu2-3,112 lys2-801 ura3-52* |  |
| NSY55 | *ypt1-1* | *MATα his3-Δ200 leu2-3,112 ura3-52*  *ypt1-T40K* | (Lipatova et al., 2013) |
| NSY1655 | LHY85, *sec12ts* | *sec12-1 ura3-52 his4 leu2-3,112 lys2-801 bar1* |  |
| NSY1656 | *pep4Δ* | NSY128 *pep4Δ::HYGRO* | This study |
| NSY1657 | *ypt1-1 pep4Δ* | NSY55 *pep4Δ::HYGRO* | This study |
| NSY1658 | *sec12ts pep4Δ* | NSY1655 *pep4Δ::HYGRO* | This study |
| NSY1547 | *SEC61-mCherry* | NSY128 *SEC61-mCherry::NAT* | (Lipatova et al., 2013) |
| NSY1548 | *ypt1-1* *SEC61-mCherry* | NSY55 *SEC61-mCherry::NAT* | (Lipatova et al., 2013) |
| NSY1659 | *sec12ts SEC61-mCherry* | NSY1655 *SEC61-mCherry::NAT* | This study |
| NSY1660 | WT *atg9Δ* | NSY128 *atg9Δ::HYGRO* | This study |
| NSY1661 | *ypt1-1 atg9Δ* | NSY55 *atg9Δ::HYGRO* | This study |
| NSY1662 | *sec12ts atg9Δ* | NSy1655 *atg9Δ::HYGRO* | This study |
| NSY1663 | *ypt1-1 vps21Δ* | NSY55 *vps21Δ::HYGRO* | This study |
| NSY1664 | *atg11Δ atg9Δ* | NSY1641 *atg11Δ::HYGRO* | This study |
| NSY1665 | *atg1Δ atg9Δ* | NSY1646 *atg9Δ::HYGRO* | This study |
| NSY1666 | *atg11Δ atg19Δ* | NSY1642 *atg11Δ::NAT* | This study |
| NSY1667 | *atg11Δ atg32Δ* | NSY1643 *atg11Δ::HYGRO* | This study |
| NSY1668 | *atg11Δ atg36Δ* | NSY1644 *atg11Δ::HYGRO* | This study |
| NSY1669 | *atg2Δ* | NSY825 *atg2Δ::KAN* | This study |
| NSY1670 | *atg18Δ* | NSY825 *atg18Δ::KAN* | This study |
| NSY1671 | WT *ire1Δ* | NSY128 *ire1Δ::KAN* | This study |
| NSY1672 | WT *hac1Δ* | NSY128 *hac1Δ::KAN* | This study |
| NSY1673 | *ypt1-1 ire1Δ* | NSY55 *ire1Δ::KAN* | This study |
| NSY1674 | *ypt1-1 hac1Δ* | NSY55 *hac1Δ::KAN* | This study |
| NSY1675 | *pep4Δ prb1Δ* | NSY1656 *prb1Δ::KAN* | This study |
| NSY1676 | *pep4Δ prb1Δ* | NSY1656 *prb1Δ::NAT* | This study |
| NSY1677 | *ypt1-1 pep4Δ prb1Δ* | NSY1657 *prb1Δ::KAN* | This study |
| NSY1678 | *ypt1-1 pep4Δ prb1Δ* | NSY1657 *prb1Δ::NAT* | This study |
| NSY1533 | *HMG1-mCherry* | NSY128 *HMG1-mCherry::KAN* | (Lipatova et al., 2013) |
| NSY1534 | *ypt1-1 HMG1-mCherry* | NSY55 *HMG1-mCherry::KAN* |  |
| NSY1544 | *HMG1-mCherry pep4Δ* | NSY1533 *pep4Δ::HYGRO* | (Lipatova et al., 2013) |
| NSY1545 | *ypt1-1 HMG1-mCherry pep4Δ* | NSY1534 *pep4Δ::HYGRO* | (Lipatova et al., 2013) |
| NSY1679 | *HMG1-mCherry pep4Δ prb1Δ* | NSY1544 *prb1Δ::NAT* | This study |
| NSY1680 | *ypt1-1 HMG1-mCherry pep4Δ prb1Δ* | NSY1545 *prb1Δ::NAT* | This study |
| NSY1681 | *SEC61-mCherry pep4Δ* | NSY1547 *pep4Δ::HYGRO* | This study |
| NSY1682 | *ypt1-1 SEC61-mCherry pep4Δ* | NSY1548 *pep4Δ::HYGRO* | This study |
| NSY1683 | *SEC61-mCherry pep4Δ prb1Δ* | NSY1681 *prb1Δ::KAN* | This study |
| NSY1684 | *ypt1-1 SEC61-mCherry pep4Δ prb1Δ* | NSY1682 *prb1Δ::KAN* | This study |
| NSY1685 | *SEC13-mCherry* | NSY128 *SEC13-mCherry::NAT* | This study |
| NSY1686 | *SEC13-mCherry pep4Δ prb1Δ* | NSY1675 *SEC13-mcherry::NAT* | This study |
| NSY1687 | *ypt1-1 SEC13-mCherry* | NSY55 *SEC13-mCherry::NAT* | This study |
| NSY1688 | *ypt1-1 SEC13-mCherry pep4Δ prb1Δ* | NSY1677 *SEC13-mcherry::NAT* | This study |
| NSY1689 | *SEC12-mCherry* | NSY128 *SEC12-mCherry::NAT* | This study |
| NSY1690 | *SEC12-mCherry pep4Δ prb1Δ* | NSY1675 *SEC12-mcherry::NAT* | This study |
| NSY1691 | *ypt1-1 SEC12-mCherry* | NSY55 *SEC12-mCherry::NAT* | This study |
| NSY1692 | *ypt1-1 SEC12-mCherry pep4Δ prb1Δ* | NSY1677 *SEC12-mcherry::NAT* | This study |
| NSY1693 | *SEC61-3xHA* | NSY128 *SEC61-3xHA::KAN* | This study |
| NSY1694 | *SEC61-3xHA pep4Δ* | NSY1656 *SEC61-3xHA::KAN* | This study |
| NSY1695 | *SEC61-3xHA pep4Δ prb1Δ* | NSY1676 *SEC61-3xHA::KAN* | This study |
| NSY1696 | *ypt1-1 SEC61-3xHA* | NSY55 *SEC61-3xHA::KAN* | This study |
| NSY1697 | *ypt1-1 SEC61-3xHA pep4Δ* | NSY1657 *SEC61-3xHA::KAN* | This study |
| NSY1698 | *ypt1-1 SEC61-3xHA pep4Δ prb1Δ* | NSY1678 *SEC61-3xHA::KAN* | This study |
| NSY1699 | *SEC13-3xHA* | NSY128 *SEC13-3xHA::KAN* | This study |
| NSY1700 | *SEC13-3xHA pep4Δ* | NSY1656 *SEC13-3xHA::KAN* | This study |
| NSY1701 | *SEC13-3xHA pep4Δ prb1Δ* | NSY1676 *SEC13-3xHA::KAN* | This study |
| NSY1702 | *ypt1-1 SEC13-3xHA* | NSY55 *SEC13-3xHA::KAN* | This study |
| NSY1703 | *ypt1-1 SEC13-3xHA pep4Δ* | NSY1657 *SEC13-3xHA::KAN* | This study |
| NSY1704 | *ypt1-1 SEC13-3xHA pep4Δ prb1Δ* | NSY1678 *SEC13-3xHA::KAN* | This study |
| NSY1705 | *SEC12-3xHA* | NSY128 *SEC12-3xHA::KAN* | This study |
| NSY1706 | *SEC12-3xHA pep4Δ* | NSY1656 *SEC12-3xHA::KAN* | This study |
| NSY1707 | *SEC12-3xHA pep4Δ prb1Δ* | NSY1676 *SEC12-3xHA::KAN* | This study |
| NSY1708 | *ypt1-1 SEC12-3xHA* | NSY55 *SEC12-3xHA::KAN* | This study |
| NSY1709 | *ypt1-1 SEC12-3xHA pep4Δ* | NSY1657 *SEC12-3xHA::KAN* | This study |
| NSY1710 | *ypt1-1 SEC13-3xHA pep4Δ prb1Δ* | NSY1678 *SEC12-3xHA::KAN* | This study |
| NSY1711 | *atg17Δ* | NSY825 *atg17Δ::KAN* | This study |
| NSY1712 | *atg11Δ atg17Δ* | NSY1499 *atg17Δ::HYGRO* | This study |
| NSY1523 | *ATG9-mCherry* | NSY825 *ATG9-mCherry::HYGRO* |  |
| NSY1527 | *ypt1-1 ATG9-mCherry* | NSY55 *ATG9-mCherry::KAN* | (Lipatova et al., 2012) |

**References**

Brachmann, C.B., Davies, A., Cost, G.J., Caputo, E., Li, J., Hieter, P., and Boeke, J.D. (1998). Designer deletion strains derived from Saccharomyces cerevisiae S288C: a useful set of strains and plasmids for PCR-mediated gene disruption and other applications. Yeast *14*, 115-132.

Jedd, G., Mulholland, J., and Segev, N. (1997). Two new Ypt GTPases are required for exit from the yeast trans-Golgi compartment. J Cell Biol *137*, 563-580.

Levi, S.K., Bhattacharyya, D., Strack, R.L., Austin, J.R., 2nd, and Glick, B.S. (2010). The yeast GRASP Grh1 colocalizes with COPII and is dispensable for organizing the secretory pathway. Traffic *11*, 1168-1179.

Lipatova, Z., Belogortseva, N., Zhang, X.Q., Kim, J., Taussig, D., and Segev, N. (2012). Regulation of selective autophagy onset by a Ypt/Rab GTPase module. Proc Natl Acad Sci U S A *109*, 6981-6986.

Lipatova, Z., Shah, A.H., Kim, J.J., Mulholland, J.W., and Segev, N. (2013). Regulation of ER-phagy by a Ypt/Rab GTPase module. Mol Biol Cell *24*, 3133-3144.
