# Supplementary material for: A Role for Macro-ER-Phagy in ER Quality Control
Source: PLoS Genet. 2015 Jul 16;11(7):e1005390. doi: 10.1371/journal.pgen.1005390 (PMC4504476; doi:10.1371/journal.pgen.1005390)
Supplement: S2 Table — (DOC) [file pgen.1005390.s010.doc]

**S2 Table. Plasmids used in this study**

| **Plasmid** | **Alias** | **Genotype** | **Source** |
| --- | --- | --- | --- |
| pNS1254 | pJC104 | 2µ, *URA3*, 4xUPRE1-crippled *CYC1* promoter-*lacZ* |  |
| pNS1407 | pRS425-GFP-*SNC1*-PEM | 2µ, *LEU2, TPI* promoter-GFP-*SNC1-*PEM |  |
| pNS180 | pRS425 | 2µ, *LEU2*, Amp^r^ |  |
| pNS181 | pRS426 | 2µ, *URA3*, Amp^r^ |  |
| pNS1191 | pFA6a-3xHA-*KanMX6* | 3xHA-*KanMX6*, Amp^r^ |  |
| pNS584 | pAG32 | *hphMX4* |  |
| pNS583 | pAG25 | *natMX4* |  |
| pNS955 | pKT127 | yEGFP-*KanMX4*, Amp^r^ |  |
| pNS1505 | pKT-mCherry | mCherry-*KanMX4*, Amp^r^ | This study |
| pNS1506 | pKT-mCherry- *NatMX4* | mCherry-*NatMX4*, Amp^r^ | This study |
| pNS1360 | p416-yEGFP-*ATG8* | *CEN*, *URA3*, *ADH1* promoter-yEGFP-*ATG8*-*CYC1* terminator |  |
| pNS1509 | p416-yDsRed (N-term) | *CEN*, *URA3*, *ADH1* promoter-yDsRed-*CYC1* terminator | This study |
| pNS1511 | p416-yDsRed-*SNC*1-PEM | *CEN*, *URA3*, *ADH1* promoter-yDsRed-*SNC1*-PEM-*CYC1* terminator | This study |
| pNS1513 | pRS426-yDsRed-*SNC1*-PEM | 2µ, *URA3, ADH1* promoter-yDsRed-*SNC1-*PEM-*CYC1* terminator | This study |
| pNS1492 | p415-yEGFP (C-term) | *CEN, LEU2,* *ADH1* promoter-yEGFP-*CYC1* terminator | This study |
| pNS1496 | p415-*SNQ2*-yEGFP | *CEN, LEU2,* *ADH1* promoter-*SNQ2*-yEGFP-*CYC1* terminator | This study |
| pNS1507 | pRS425-*SNQ2*-yEGFP | 2µ, *LEU2, ADH1* promoter-*SNQ2-*yEGFP-*CYC1* terminator | This study |

**References**

Cox, J.S., Shamu, C.E., and Walter, P. (1993). Transcriptional induction of genes encoding endoplasmic reticulum resident proteins requires a transmembrane protein kinase. Cell *73*, 1197-1206.

Goldstein, A.L., and McCusker, J.H. (1999). Three new dominant drug resistance cassettes for gene disruption in Saccharomyces cerevisiae. Yeast *15*, 1541-1553.

Lipatova, Z., Belogortseva, N., Zhang, X.Q., Kim, J., Taussig, D., and Segev, N. (2012). Regulation of selective autophagy onset by a Ypt/Rab GTPase module. Proc Natl Acad Sci U S A *109*, 6981-6986.

Lipatova, Z., Shah, A.H., Kim, J.J., Mulholland, J.W., and Segev, N. (2013). Regulation of ER-phagy by a Ypt/Rab GTPase module. Mol Biol Cell *24*, 3133-3144.

Longtine, M.S., McKenzie, A., 3rd, Demarini, D.J., Shah, N.G., Wach, A., Brachat, A., Philippsen, P., and Pringle, J.R. (1998). Additional modules for versatile and economical PCR-based gene deletion and modification in Saccharomyces cerevisiae. Yeast *14*, 953-961.

Sheff, M.A., and Thorn, K.S. (2004). Optimized cassettes for fluorescent protein tagging in Saccharomyces cerevisiae. Yeast *21*, 661-670.

Sikorski, R.S., and Hieter, P. (1989). A system of shuttle vectors and yeast host strains designed for efficient manipulation of DNA in Saccharomyces cerevisiae. Genetics *122*, 19-27.
